# Demographic history of the Jomon people: insights from whole-mitogenome analysis

**DOI:** 10.1537/ase.251024

**Published:** 2026-01-30

**Authors:** Koki Yoshida, Yoshiki Wakiyama, Yuka Nakamura, Guido Valverde, Akio Tanino, Daisuke Waku, Takafumi Katsumura, Motoyuki Ogawa, Tomohito Nagaoka, Kazuaki Hirata, Kae Koganebuchi, Yusuke Watanabe, Jun Ohashi, Minoru Yoneda, Ryuzaburo Takahashi, Hiroki Oota

**Affiliations:** 1 Department of Biological Sciences, Graduate School of Science, The University of Tokyo, Tokyo, 113-0033 Japan; 2 Department of Anatomy, School of Medicine, Kitasato University, Kanagawa, 252-0374 Japan; 3 Institute for Mummy Studies, Eurac Research, Bolzano, Viale Druso 1, 39100 Bolzano, Italy; 4 Department of International Agricultural Development, Faculty of International Agriculture and Food Studies, Tokyo University of Agriculture, Tokyo, 156-8502 Japan; 5 Department of Anatomy, St Marianna University School of Medicine, Kanagawa, 216-8511 Japan; 6 Faculty of Management and Economics, Aomori Public University, Aomori, 030-0196 Japan; 7 The University Museum, The University of Tokyo, Tokyo, 113-0033 Japan; 8 Faculty of Letters, Arts and Sciences, Waseda University, Tokyo, 169-8050 Japan

**Keywords:** Jomon, ancient DNA, whole-mitogenome sequence, demographic history, simulation

## Abstract

The Jomon culture that spread across the Japanese archipelago began about 16000 years ago and lasted for over 10000 years. The population history of the Jomon people, prehistoric hunter-gatherers bearing the Jomon culture, is of great interest in understanding prehistoric East Eurasians. Traditionally, population size and its fluctuations, i.e. Jomon demography, have been estimated in the archaeological context, but over the past 20 years, statistical methods using genome sequence data have been sufficiently developed. To investigate their demography, we determined the complete whole-mitochondrial genome (mitogenome) sequences from 13 Jomon individuals and conducted population genetic analysis on 40 Jomon mitogenomes, including previously published data. By simulation, we showed that east–west frequency differences between two haplogroups typical of the Jomon people, N9b and M7a, could be caused by a genetic drift under conditions of a small initial effective population size, an extreme population split, and limited migration between the eastern and western populations, suggesting that the regionally unbalanced haplogroup distribution does not necessarily contradict the monophyletic origin scenario of the Jomon people implied by recent nuclear genome analyses. We found an effective population size (*N*_e_) increase during the Incipient–Initial phase of the Jomon period, which had not been observed in analyses of mitogenome sequences from present-day Japanese populations. This endemic demographic pattern is pronounced in the eastern part of the archipelago, under the assumption of no gene flow between the Eastern and Western Jomon. This study sheds light on the demography of the Jomon people and shows an alternative scenario of the Jomon peopling history estimated based on whole-mitogenome data.

## Introduction

The Jomon people are prehistoric hunter-gatherers who inhabited the Japanese archipelago, located at the eastern end of the Eurasian continent. They are characterized by the earliest use of pottery adorned with cord-marking patterns, from which the name “Jomon” is derived, and a sedentary lifestyle, which is uncommon among hunter-gatherers (Habu, 2004). The Jomon period is divided into six phases based on temporal changes in the style of pottery: Incipient (c. 16500–11300 years ago (ya)), Initial (c. 11300–7100 ya), Early (c. 7100–5400 ya), Middle (c. 5400–4400 ya), Late (c. 4400–3200 ya), and Final (c. 3200–2400 ya) (Habu, 2004). Thus, the Jomon period spanned over 10000 years.

During the long Jomon period, how did the population sizes change over time? In addressing this question, Koyama (1978) estimated population sizes based on the number of archaeological sites per region and on documentary evidence from the historical period. The results suggest gradual population growth across the Japanese archipelago from the Initial to the Early phase, reaching its peak during the Middle phase, with the eastern part of the archipelago (corresponding to the Kanto region mainly) gaining a larger population size than the western part, employing the Chubu–Kinki boundary as the east–west border. While several studies on the Jomon population have been published since Koyama’s estimation, a comprehensive analysis covering the entire demographic history of the Jomon people across the Japanese archipelago remains limited. Koyama (1978) continues to serve as a primary reference, underscoring the need for more extensive and updated research that fully incorporates recent archaeological and genetic findings.

More recently, Bayesian skyline plot (BSP) (Drummond et al., 2005) analysis has been applied to multiple whole-mitogenome nucleotide sequences from present-day individuals in the Japanese archipelago with the aim of estimating the prehistoric demography (Jinam et al., 2021; Mizuno et al., 2021). These previous BSPs show rapid growth in effective population size (*N*_e_) after the end of the Jomon period and with the beginning of the Yayoi period, coinciding with the advent of wet rice cultivation. However, since the present-day Japanese gene pool includes mitogenomes from indigenous Jomon people and continental migrants who arrived about 3000 ya (Hanihara, 1991; Horai et al., 1996; Omoto and Saitou, 1997), BSP analysis based on them would reflect both Jomon and migrant demographic changes. The difficulty in identifying mitogenomes specific to the Jomon people from the present-day Japanese gene pool makes it impossible to observe the demographics of the Jomon population alone (Watanabe et al., 2019). On the other hand, the situation is different for Y-chromosomal DNA. Watanabe et al. (2019) conducted a phylogenetic analysis of the East Asian Y chromosome and identified a major clade (comprising 35.4% of Hondo Japanese) exclusively composed of present-day Japanese people, likely to be derived from the Jomon people. BSP analysis using these Y chromosomes indicated a notable decline in the male population around 2500 ya, aligning with the transition from the end of the Jomon to the early Yayoi period. However, this study is based on present-day Japanese genomes; thus, sequences specific to the Jomon people cannot be truly identified without direct analysis of the Jomon people.

Genetic studies surrounding regional disparities between eastern and western Jomon populations have predominantly centered on the frequency of mitochondrial haplogroups. Among the Jomon people across the Japanese archipelago, three prominent haplogroups—M7a, N9b, and D4b2—have been identified (Shinoda and Kanai, 1999; Adachi et al., 2011). Although M7a and N9b exhibit low frequencies in the current Japanese archipelago, several lines of previous studies have shown that almost all Jomon individuals belong to one of these haplogroups: N9b predominates among eastern Jomon, while M7a prevails among western counterparts. In the broader perspective of N9 and M7, both are distributed in the coastal regions of East Eurasia, but N9 is mainly found in Northeast Asia including Primorsky Krai (Yao et al., 2002; Starikovskaya et al., 2005; Lee et al., 2006), while M7 is mainly found in Southeast Asia and southern China (Kivisild et al., 2002; Trejaut et al., 2005; Hill et al., 2007). This regionally skewed distribution of both haplogroups suggests two possible routes taken by the Jomon ancestral population migrating to the archipelago in the Upper Paleolithic period, one from the north, represented by N9b, and one from the west, represented by M7a (Tanaka et al., 2004; Adachi et al., 2011; Shinoda, 2019). If we follow this scenario, the east–west frequency differences between N9b and M7a would have been caused by the multiple migration waves, implying that the Jomon were formed by admixture of distinct lineage groups. It should be noted, however, that one of the main reasons for this scenario is the distribution of haplogroups in present-day populations in East Eurasia because the genetic diversity in present-day populations has been affected by migration and admixture over the past tens or thousands of years, not necessarily preserving past genetic diversity.

Recent studies based on sequence data obtained by high-throughput sequencing (what we call next-generation sequencing, NGS) have not necessarily supported this scenario (Watanabe et al., 2024; Duggan et al., 2013; Mizuno et al., 2020, 2023; Gakuhari et al., 2020; Cooke et al., 2021; Jeong et al., 2023). The whole-mitogenome sequence data show that N9b subtypes found in the Jomon and the present-day Japanese people differ from those found in the putative source populations from continental regions (Duggan et al., 2013; Mizuno et al., 2020, 2023). The nuclear genome sequence data of the Jomon genome have shown that Jomon individuals exhibit a high homogeneity within the diversity of East Eurasian populations and diverge deeply from other East Asian populations (Gakuhari et al., 2020; Cooke et al., 2021; Jeong et al., 2023). A more recent nuclear whole-genome analysis of 41 Jomon individuals suggested that they formed a single clade in the phylogenetic tree, within which they segregate according to a single geographic gradient (Watanabe et al., 2024). Simply interpreted, these data suggest an alternative scenario: the ancestral population of the Jomon people was of a single lineage, which may have initially settled in part of the archipelago during the Upper Paleolithic period. That is, the east–west frequency difference between haplogroups N9b and M7a could have been formed by genetic drift associated with the spread of the single ancestral population of the Jomon people. This scenario, however, remains untested because the whole-genome data of the Jomon people have only become available within the last decade.

Analyses of population genetics to examine demographic history over thousands of years ideally require more than 50 individuals (Hedrick, 2009). However, it has been difficult to achieve this standard using ancient genomes because there are far fewer samples available for analysis compared with present-day genomes. Nevertheless, the recent accumulation of whole-mitogenome sequences from Jomon bones has opened the door for the estimation of population demography using a relatively large number of Jomon individuals. In this study, we investigate the demographic history of the Jomon people using their whole-mitogenome sequence data. We have extracted DNA from 52 human remains excavated from Jomon sites and determined 13 new complete whole-mitogenome sequences. Combining previously published mitogenome sequence data, we have constructed a 40-individual dataset and conducted population genetic analyses. We found that the east–west frequency differs between haplogroups N9b and M7a on a complete whole-mitogenome sequence basis, and estimated that N9b and M7a appeared about 23000 and 26000 ya, respectively. Our frequency-based simulation has demonstrated that that large frequency differences can arise solely due to genetic drift, especially under conditions of a small initial effective population size, an extreme population split, and limited migration between eastern and western populations. The results of the demographic analyses show an increase in *N*_e_ between the Incipient and Initial phases of the Jomon period that is not observed in contemporaneous continental East Asian peoples. Notably, this population composition was more pronounced in the eastern part of the Japanese archipelago than in the western part if we assume that there was no gene flow between east and west, suggesting that the Jomon population exhibited significant regional demographic diversity.

## Material and Methods

### Archaeological samples

Fifty-two pieces of skeletal remains excavated from three archaeological sites, Gionbara (GB), Kikumatenaga (KT), and Saihiro (SH) shell mounds, located in Ichihara City, Chiba Prefecture, were examined in this study (Supplementary Table S1). The GB and SH shell mounds were assigned to the Late–Final phases of the Jomon period (c. 4400–2400 ya) and the KT shell mound to the Late phase of the Jomon period (c. 3200–2400 ya). The results of radioisotope dating are described in Nakamura et al. (2024). Hereinafter, the three sites will collectively be referred to as the Ichihara Jomon sites.

### Radiocarbon dating

Radiocarbon dating was conducted using accelerator mass spectrometry (AMS) at The University Museum, The University of Tokyo. Calibration of dates was performed with reference to IntCal20 and Marine20 (Reimer et al., 2020; Heaton et al., 2020) and calibrated ages were determined using OxCal 4.4 software (Ramsey, 2009). Correction for the marine reservoir effect, which causes apparent radiocarbon dates to appear older due to seafood consumption, was achieved by assessing the marine carbon effect based on δ^13^C values. The marine contribution was estimated by utilizing mean δ^13^C values (–22.6‰ for land mammal bones and –10.9‰ for marine fish bones) derived from shell middens in Chiba Prefecture, assuming a 5% margin of error. A regional correction value (Δ*R*) for Tokyo Bay (–98 ± 37 years) was applied to Marine20 based on the analysis of a shell collected in 1882 AD (534 ± 36 BP) (Yoshida et al., 2010). The median of the probability distribution of dating was used as the estimated age.

### DNA extraction

All ancient DNA experiments were performed in clean rooms built exclusively for ancient DNA analyses and installed in the Department of Biological Sciences, Graduate School of Science, The University of Tokyo or in the Department of Anatomy, Kitasato University School of Medicine. All bones and teeth were exposed to ultraviolet (UV) light for 15 minutes on either side to reduce DNA contamination from the sample surfaces. Because a sample (GB2-A1a) was calcified and found to be difficult to extract DNA, any further operations were not performed. Petrous bone was cut with an UV-irradiated diamond disc cutter (SHOFU), and a wedge section of the otic capsule region, including the ‘C-part’ in Pinhasi et al. (2015), was targeted for sampling. The tooth sample was cut with the UV-irradiated diamond disc cutter and separated into crown and root. The dentin powder was collected by an UV-irradiated drill (SHOFU). Between 5 and 150 mg of bone pieces or powder was collected for DNA extraction.

DNA was extracted following the methods of previous studies (Gamba et al., 2014; Damgaard et al., 2015; Gakuhari et al., 2020). A piece of bone piece or bone powder was incubated in a 5-ml DNA LoBind tube (Eppendorf) with 2 ml of lysis buffer (Tris–HCl pH 7.4, 20 mM; Sarkosyl NL, 0.7%; EDTA pH 8.0, 47.5 mM; Proteinase K, 0.65 U/ml) for 15 min at 50°C at 900 r.p.m. in a Thermomixer (Eppendorf). The samples were centrifuged at 2300 *g* for 10 min, and the supernatant was discarded. Then, 2 ml of fresh lysis buffer was added to the tubes and the mixture incubated overnight (>16 h) at 60°C at 900 r.p.m. After digestion, the tubes were centrifuged at 2300 *g* for 10 min. About 2 ml of supernatants was then transferred to ultrafiltration tubes (Amicon Ultra-4 Centrifugal Filter Unit 10K, Merck). Then, 2 mL of TE buffer was added, and the samples were centrifuged at 2300 *g* until the final concentrations reached 100 μl. These concentrates were then transferred to a silica column (MiniElute PCR Purification Kit, QIAGEN) and purified according to the manufacturer’s instructions, except for the elution step with 60 μl EBT buffer (EB buffer with 0.05% Tween 20 at the final concentration) preheated to 60°C. The concentration of the DNA extract was measured using a Qubit 4 Fluorometer (Invitrogen). The DNA fragment length distribution was measured using a Bioanalyzer (Agilent) or a TapeStation (Agilent).

### Library preparation

Double-stranded DNA libraries were prepared with NEBNext Ultra II DNA Library Prep Kit for Illumina and NEBNext Multiplex Oligos for Illumina (96 unique dual-index primer pairs) (New England Biolab (NEB)). Around 1 ng of DNA was used for each library. Libraries were constructed according to the manufacturer’s protocol, except for the following two points: the adapter was diluted 10-fold, and size selection was performed. In the size selection, 90 μl of Agencourt AMPure XP (Beckman Coulter) was added to the reaction, and beads with long fragments (>150 bp) were removed by transferring the supernatant to the new 1.5-ml tube. Another 90 μl of AMPure XP solution was added to the supernatant, after which the short fragments were recovered.

For four samples (KT2, KT68, SH7-3, and SH7-4), uracil-DNA glycosylase (UDG)-treated libraries (Rohland et al., 2015) were prepared. First, 5 ng of DNA was added to the treatment solution (10× Tango buffer, 5 μl; 2.5 mM dNTP, 0.2 μl; 100 mM ATP, 0.5 μl; 10 U/μl T4 polynucleotide kinase, 2.5 μl; 1000 U/μl USER Enzyme, 3 μl; ultrapure water up to 50 μl in total). The reaction solution was incubated in a thermal cycler at 37°C for 3 h. Then, 1 μl of T4 polymerase was added and the mixture incubated at 25°C for 15 min followed by 12°C for 5 min. The product was purified using a Mini Elute PCR purification kit (Qiagen) and extracted twice with EBT buffer warmed to 60°C (first extraction, 30 μl; second extraction, 23 μl), then finally about 50 μl of UDG-treated DNA was obtained; 25 μl of this UDG-treated DNA was used for library preparation.

### High-throughput sequencing

Small-scale sequencing was performed on MiSeq (Illumina) to assess the ratios of endogenous human DNA and postmortem damage patterns. We mapped the reads to the human reference genome sequence (hg19) and calculated a mapping rate (MR), defined as the number of mapped reads divided by the total number of reads. Thirteen individuals that exhibited a MR exceeding 15% (see Supplementary Table S1) were subsequently sequenced on HiSeq or NovaSeq (Illumina) with 150-bp or 100-bp paired-ends at the National Institute of Genetics, AZENDA, or MACROGEN.

### Raw data processing

The FASTQ files were processed using the following pipeline. For paired-end sequence reads, we used AdapterRemoval v. 2.3.2 (v. 2.2.2 for screening) (Schubert et al., 2016) to remove ambiguous and low-quality bases at ends of reads (--trimns and --trimqualities), and short reads (--minlength 35), combining them into consensus sequence (-collapse). For single-end sequence reads, we used cutadapt v. 4.4 (Martin, 2011) with options (--trim-n, --quality 2, --length 35) corresponding to those of AdapterRemoval. Using BWA v. 0.7.17 (Li and Durbin, 2009), the trimmed reads were mapped to the revised Cambridge Reference Sequence (rCRS). To map the reads on the end of the reference, we used circularmapper v. 1.93.5 (Peltzer et al., 2016). BAM files from the same library were merged into a BAM file using SAMtools v. 1.6 (v. 1.10 for screening) (Li et al., 2009). CleanSam and MarkDuplicates in Picard Tools v. 3.0.0 (v. 2.21.8 for screening) (https://broadinstitute.github.io/picard/) were used to soft-clip beyond-end-of-reference alignments and remove duplicate reads, respectively. The mapping rate was calculated using flagstat in SAMtools. The misincorporation patterns were checked by mapDamage2 v. 2.2.0 (v. 2.0 for screening) (Jónsson et al., 2013) and hard-clipped two or ten bases of the 5' end and ten bases of the 3' end, where substitutions occur frequently, using TrimBam v. 1.0.15 in BamUtil (Jun et al., 2015). For UDG-treated samples, two bases of 3' and 5' end of reads were hard-clipped. After merging BAM files from the same individual into a BAM file, mapped reads with mapping quality below Phred score 30 were removed using SAMtools v. 1.6. We calculated the average depth using DepthOfCoverage in the Genome Analysis Toolkit (GATK) v. 4.3 (Mckenna et al., 2010). VCF files were obtained using HaplotypeCaller in GATK and converted to the complete whole-mitogenome sequences in FASTA format using FastaAltenateReferenceMaker in GATK. We applied the same processing pipeline for published ancient genomes in FASTQ or BAM format. The contamination rate was estimated using MitoSuite v. 1.0.9 (Ishiya and Ueda, 2017). The haplogroup was called by HaploGrep 3 (Schönherr et al., 2023).

### Previously published data

**Figure 1.**
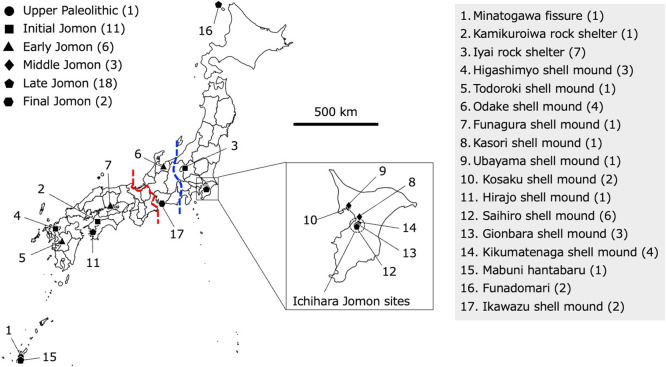
Archaeological sites from which the ancient samples were examined in this study. Each site is marked with a circle, square, triangle, rhombus, pentagon, and hexagon, representing the Upper Paleolithic period, and the Initial, Early, Middle, Late, and Final phases of the Jomon period, respectively. The square shows an enlarged view of Chiba Prefecture, where the 13 individuals we newly sequenced were excavated. The red and blue dashed lines indicate the Chubu–Kinki boundary and the Itoigawa–Shizuoka Tectonic Line, respectively.

Whole-mitogenome sequences from 27 Jomon individuals (11 Initial, 6 Early, 3 Middle, 5 Late, 2 Final) previously published were used for comparative analyses (Figure 1, Table 1). These samples were excavated from archaeological sites across three regions of the Japanese archipelago: Hokkaido (2 samples), Hondo—comprising Honshu, Shikoku, and Kyushu (24 samples), and Ryukyu (1 sample) (Supplementary Figure S1). With the addition of our newly sequenced Late Jomon individuals, we obtained a total of 40 whole-mitogenome sequences from Jomon individuals: 11 from the Initial, 6 from the Early, 3 from the Middle, 18 from the Late, and 2 from the Final periods (Table 1). In addition, one Upper Paleolithic individual from Ryukyu (Minato 1) and one present-day individual from Sudan in Northeast Africa (JN655840.1) were included in the phylogenetic analysis (Table 1).

We aligned all FASTA-formatted sequences to the Cambridge Reference Sequence (rCRS) using MUSCLE program (Edgar, 2004) in MEGA X (Kumar et al., 2018). As neutral regions, we used non-coding regions, i.e. nucleotide positions 1–576 and 16024–16569 of the rCRS, which includes hypervariable regions I and II (1094 bp). Additionally, a concatenation of 13 protein-coding gene sequences (11341 bp) was used as a coding region. Due to alignment challenges caused by repetitiveness and indel, nucleotide positions 303–315, 522–523, 16180–16193, and 16519 were excluded from the dataset for the subsequent analyses.

### Phylogenetic analyses

To examine the east–west frequency differences between haplogroups N9b and M7a observed on the basis of sequences of whole-mitogenome, we constructed a median-joining network (Bandelt et al., 1999) using POPART v. 1.7 (Leigh et al., 2015). An Upper Paleolithic and a present-day individual were added as outgroups to root the tree (Table 1).

We then estimated the divergence time of the most recent common ancestor (MRCA) of each haplogroup using BEAST v. 2.7.5 (Bouckaert et al., 2019). Input XML files for BEAST were generated by the BEAUTi program, assigning tip dates as listed in Table 1. For individuals lacking precise calibration dates, we referred to their subperiod or used dates of other individuals from the same archaeological site. We selected General Time Reversible model as the substitution model, which is a type of gamma-site model. The optimized relaxed clock was applied with molecular clock rate (per site per year) of 2.77 × 10^–8^ for the whole-mitogenome and 1.57 × 10^–8^ for the coding region (Fu et al., 2013; Posth et al., 2016). We used a Yule model as the prior for the coalescent tree and incorporated previously reported divergence times for specific haplogroups assuming a normal distribution: 60000 ya (*σ* = 10000) for haplogroups M and N; 40000 ya (*σ* = 5000) for haplogroup D; 20000 ya (*σ* = 5000) for haplogroup N9 (Tanaka et al., 2004; Derenko et al., 2010; Soares et al., 2012). We used a neighbor-joining starting tree constructed in MEGA X with the *p*-distance as the genetic distance (Kumar et al., 2018).

For the Markov chain Monte Carlo (MCMC) analyses, we set a chain length of 1 × 10^8^ with 1 × 10^6^ burn-in steps to obtain adequate samples for parameter estimation. XML files were modified following BEAST’s online tutorial and input into BEAST for analysis. After running BEAST, the output tree files were annotated using TreeAnnotator in the BEAST packages and visualized with FigTree v. 1.4.4 (Rambaut, 2018).

### Haplogroup frequency simulation

To investigate whether genetic drift could increase the frequency differences of haplogroups between eastern and western populations, we conducted simulations based on the Wright–Fisher model (Wright, 1931). In these simulations, we assumed that the Jomon and their ancestral populations were random mating populations consisting of N9b and M7a. Based on our divergence time estimation, we set the temporal range to 1000 generations (0th generation = 25000 years ago), where the generation time is 25 years. We assumed the first 100 generations as a period of the common ancestral population (CAP), and took constant *N*_e_ to be 1000, 5000, and 10000. The lower limit of *N*_e_ = 1000 is based on that estimated from the whole-genome analyses of the Jomon people (Cooke et al., 2021), while the upper limit of *N*_e_ = 10000 is set according to the archaeological estimate based on the number of archaeological sites, which suggests the actual population size in the Japanese archipelago during the Upper Paleolithic period was at most 10000 (Koyama, 1992). The frequencies of the two haplogroups started at 0.5 and changed by genetic drift during the CAP period. At the 100th generation (22500 ya), CAP was divided into two populations, which were assumed to be western and eastern populations. The population split ratio patterns were 1:9, 2:8, 3:7, 4:6, and 5:5, vice versa. The post-split demographic trajectories were modeled using population size estimates based on the number of archaeological sites. Specifically, we applied the Chubu–Kinki boundary as the boundary between eastern and western Japan and plotted the effective population size of each phase of the Jomon period by assuming one-tenth of the actual population size estimated by Koyama and Sugito (1984). All plots, including *N*_e_ at the population split, were approximated by a sigmoid curve based on the least-squares method. In addition to the *N*_e_, we incorporated two parameters: migration rate and migration interval. The migration rate, defined as the proportion of individuals migrating to the other population relative to the *N*_e_ of that generation, was set at 0%, 0.1%, or 1%. The migration interval, defining how often migration events occurred, was set at 1, 10, or 100 generations. We ran 1000 simulation trials to examine the probability that the frequency difference (east minus west) for one haplogroup, assumed to be N9b, would exceed the approximate observed difference of 0.5. We assessed the probabilities at 100-generation increments between the 400th and 900th generations, corresponding to the Jomon period in this model. This approach allowed us to explore whether genetic drift under various demographic and migration conditions could plausibly account for the large observed haplogroup frequency differences between eastern and western Jomon people.

### Genetic diversity statistics

Using DnaSP v. 6.12.03 (Rozas et al., 2017), we calculated the values of genetic diversity statistics, nucleotide diversity, and Tajima’s *D* for the non-coding regions of the mitogenome. Nucleotide diversity is the mean pairwise difference of nucleotides divided by the sequence length and indicates the genetic diversity within a population (Nei, 1987). Tajima’s *D* evaluates the difference between nucleotide diversity and the proportion of polymorphic sites adjusted for the sample size (Tajima, 1989). Although Tajima’s *D* was originally developed for testing the neutral hypothesis in molecular evolution, this statistic can be used to detect the change in population size when targeting neutral regions, since it is based on the expectation of a constant population size at mutation-drift equilibrium (Aris-Brosou and Excoffier, 1996; Oota et al., 2002a; Mousset et al., 2004; Matsukusa et al., 2010; Katsumura et al., 2012; Puzey et al., 2017; Flanagan et al., 2021; Peng et al., 2023). Both statistics were calculated for the 40 Jomon individuals or divided into groups, as well as for 103 CHB individuals (Chinese from Beijing) and 104 JPT individuals (Japanese from Tokyo) in phase 3 (1000 Genomes Project Consortium, 2015).

### BSP to estimate demographic history

The BSP plot is a population-size piecewise constant model that can be adapted to a wide range of demographic scenarios (Pybus et al., 2000). It is useful as a model selection tool to indicate the most appropriate demographic model for any given dataset (Pybus et al., 2002). To investigate the change in *N*_e_ of the Jomon population, we employed BSP analysis (Drummond et al., 2005) using BEAST v. 2.7.5 (Bouckaert et al., 2019). BSP is based on Bayesian coalescent inference of multiple haploid genomes experiencing uniparental inheritance without recombination. The tip dates, the substitution model, and molecular clock rates were set the same as the divergence time estimation. Following a previous study, we applied a strict clock model (Jinam et al., 2021), MCMC was set to the chain length of 1 × 10^7^ with 1 × 10^5^ burn-in steps to collect sufficient samples for parameter estimation. After the analyses by BEAST, we put the output log files into Tracer v. 1.7.2 (http://tree.bio.ed.ac.uk/software/tracer/) to draw BSP.

## Results

### Data quality of the newly sequenced individuals

We obtained DNA from 51 pieces of skeletal material at the Ichihara Jomon sites. The MR, the rate of reads to the human reference genome out of raw sequence reads, was between 0% and 62.97%, and 13 individuals exceeded 15% (see Supplementary Table S1). We further sequenced these 13 individuals and obtained complete whole mitogenome sequences with average depths of 20- to 270-fold and contamination rates below 2% (Table 1). Filtered reads showed deamination only at the 3' end (see Supplementary Figure S2), which is a characteristic damage pattern when using NEBNext Ultra II DNA Library Prep Kit as previously reported (e.g. Ning et al., 2019).

### Distribution and divergence time of the major haplogroups

**Figure 2.**
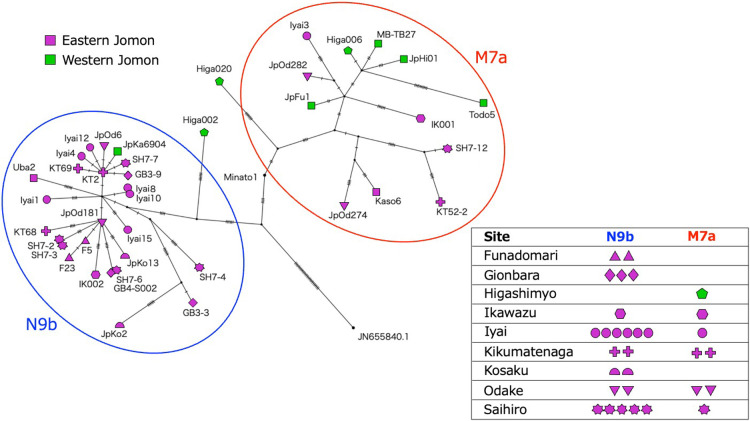
The phylogenetic network of whole-mitogenome sequences using the median-joining method. The Jomon individuals were assigned to eastern (magenta) or western (green) groups based on the Chubu–Kinki boundary suggested by Koyama (1978). Each mark, except for squares, represents an archaeological site that includes multiple individuals, whereas squares represent a site that includes only a single individual. Among the 40 mitogenome sequences, the pairs of sequences from six individuals were identical to each other. The three pairs are described as SH7-2/SH7-3, SH7-6/GB4-S002, and Iyai8/Iyai10. The number of hatch marks on each branch is the number of mutations. The box on the right shows the number of individuals belonging to haplogroups N9b or M7a at sites where multiple individuals were used in this study. There is a single individual from Higashimyo in M7a, because Higa002 and Higa020 did not belong to either N9b or M7a, although three individuals from Higashimyo were examined in this study.

To confirm whether the east–west frequency differences between haplogroups N9b and M7a are observed based on whole-mitogenome sequences, we constructed a phylogenetic network using 40 Jomon individuals (Figure 2). The network showed two prominent clades corresponding to haplogroups N9b and M7a, encompassing 26 and 12 individuals, respectively. Consistent with findings of a previous study (Mizuno et al., 2023), only two (Higa002 and 020 from the Initial phase) out of the 40 Jomon individuals, along with the Upper Paleolithic Minatogawa individual (Minato1), did not fall into the two major haplogroups. The 38 Jomon individuals belonging to either N9b or M7a haplogroups were divided into the eastern and western groups by the Chubu–Kinki boundary. Of the 32 Eastern Jomon mitogenomes, 25 belonged to N9b, while 5 out of 8 Western Jomon mitogenomes belonged to M7a. All 13 individuals from the Ichihara Jomon sites, classified as part of the eastern Jomon population, belonged to either the N9b or M7a haplogroups, with 11 individuals belonged to N9b, and only 2 to M7a. These results are consistent with previous studies on the Jomon mitogenome haplogroups, which indicated a higher prevalence of N9b in the eastern region of the archipelago and M7a in the western region (Adachi et al., 2008, 2013, 2021; Mizuno et al., 2023).

To verify this east–west dichotomy in haplogroup distribution using the whole-mitogenome sequences, we performed a Fisher’s exact test for 38 individuals and detected a significant association of N9b with eastern Jomon individuals and M7a with western Jomon individuals (*P* < 10^–5^). We categorized the 31 individuals from nine sites into either N9b or M7a and found four sites detected only either N9b or M7a, while five sites detected both haplogroups (Figure 2). Thus, while these haplotypes were not strictly confined to specific regions, there existed statistically significant disparities in the frequency distribution of N9b and M7a between the eastern and western populations.

Examining the phylogenetic network’s topology, we observed star-like structures, where multiple sequences diverged from a single sequence, within the N9b clade but not the M7a clade. The presence of such star-like structures typically indicates rapid population expansion (Di Rienzo and Wilson, 1991; Oota et al., 2002b). This suggests that a rapid demographic expansion occurred within the lineage of N9b or within the eastern Jomon population (as opposed to the western population).

To investigate the time when haplogroups N9b and M7a emerged, we estimated the divergence time of the root of each clade in the phylogenetic network. Using the whole-mitogenome, divergence times were estimated at approximately 23000 ya (95% CI: 19611–31213 ya) for N9b and 26000 ya (95% CI: 21520–35959 ya) for M7a (Supplementary Figure S3A). Similar estimates were obtained using coding regions with adjusted mutation rates (Supplementary Figure S3B). These dates correspond to the Upper Paleolithic period; however, it remains uncertain whether these haplogroups emerged on the East Eurasian continent or within the Japanese archipelago. The lack of a substantial difference in divergence times between N9b and M7a suggests that they may have entered the Japanese archipelago as part of a single migration event.

**Figure 3.**
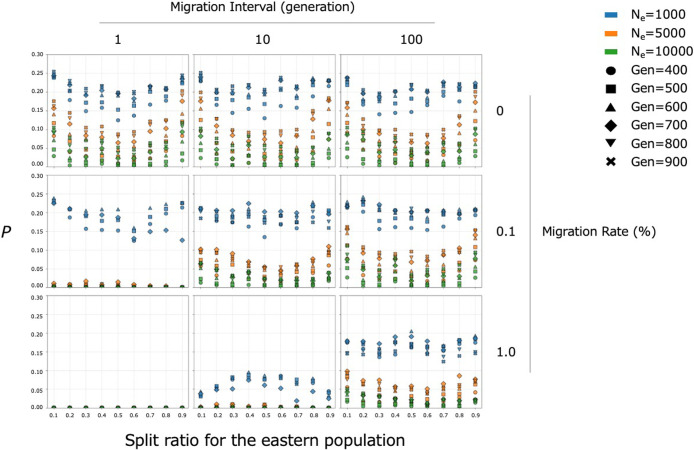
The probabilities (*P*) that the frequency difference (east–west) for one haplogroup, assumed to be N9b, would reach 0.5 or greater. Each box corresponds to a different combination of migration rates (0%, 0.01%, 0.1%) and intervals (1, 10, 100 generations). In each box, the split ratio for the eastern population is shown on the *x* axis and the *P* on the *y* axis. Note that the maximum on the *y* axis is 0.30. Each color represents the initial effective population size before the population split, and each mark represents 100-generation increments between the 400th and 900th generations, corresponding to the Jomon period in this model.

We subsequently conducted simulations to investigate whether the east–west frequency difference between these haplogroups could be increased by genetic drift under the assumption that the Jomon people originated from a common ancestral population. Figure 3 shows the probabilities (*P*) that the frequency difference (east–west) for one haplogroup, assumed to be N9b, would reach 0.5 or greater. The results of our simulation indicate the following trends. First, a smaller initial effective population size is associated with a higher *P*. Second, more extreme population split ratios tend to result in higher *P*. An exception to this trend was observed under the specific condition of *N*_e_ = 1000, the migration rate of 1%, and the migration interval of 10 years, where more extreme split ratios led to lower *P*. Third, the effect of migration on *P* was found to depend on the migration rate per generation, defined as the migration rate divided by the migration interval; the smaller this value, the greater the resulting *P*. Moreover, *P* tended to increase over generations when the migration rate per generation was low. In summary, these patterns suggest that even if a single ancestral population is the origin, large east–west haplogroup frequency differences could arise under the following conditions: (1) a small initial effective population size, (2) an extreme population split, and (3) limited migration between the two split populations. Under all of these conditions, *P* reached close to 30%.

### Demographic change in the Jomon period

**Figure 4.**
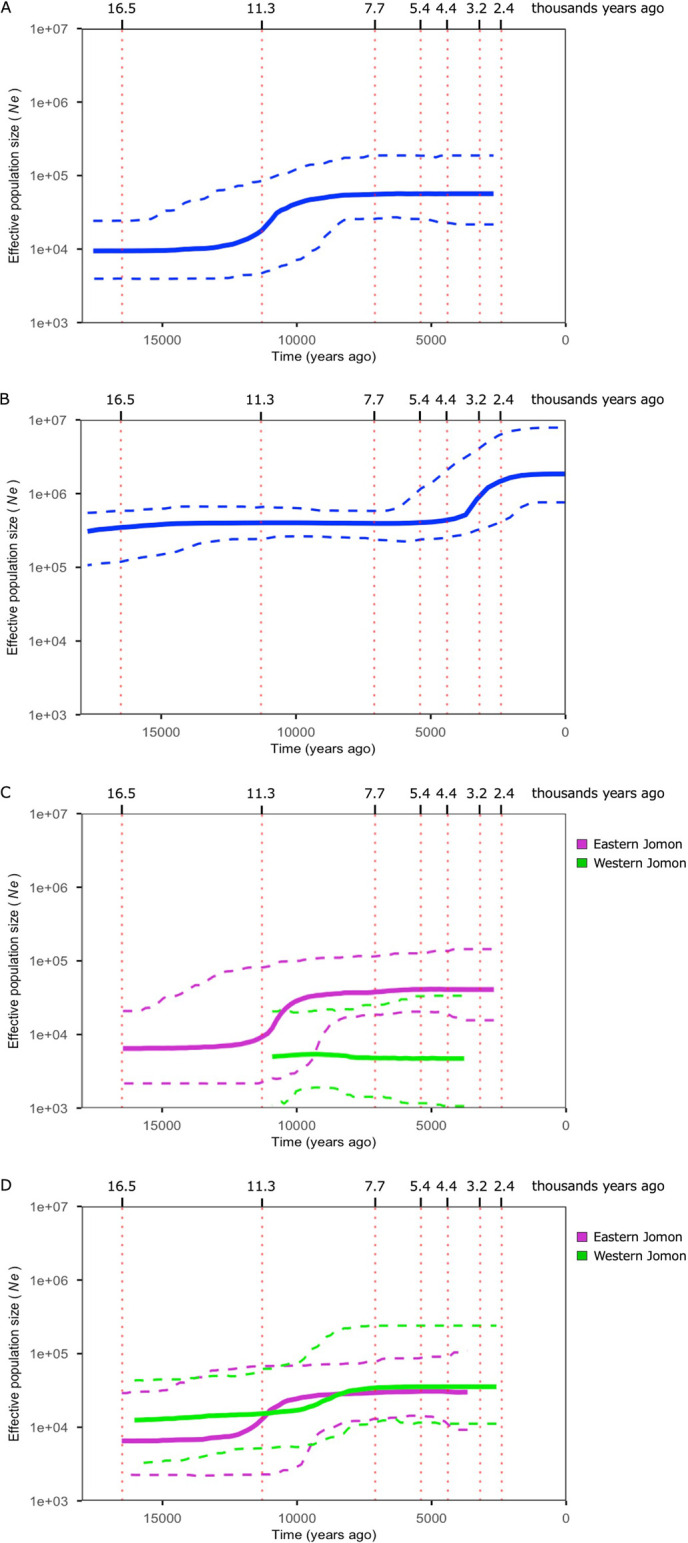
Bayesian skyline plot of all Jomon individuals (A), present-day Japanese individuals (B), and eastern (magenta) and western (green) Jomon individuals using the Chubu–Kinki boundary (C) and the Itoigawa–Shizuoka Tectonic Line (D) as the east–west boundary of the Japanese archipelago. Time on the *x* axis is shown in years ago, and *N*_e_ on the *y* axis is shown on a log scale. The bold line indicates the estimated median *N*_e_, the two dashed lines across it indicate 95% CIs, and the dotted line parallel to the *y* axis indicates the boundary between the six phases of the Jomon period, i.e. Incipient, Initial, Early, Middle, Late, and Final.

To assess changes over time in the *N*_e_, we conducted BSP analyses using whole-mitogenome sequences. In the BSP encompassing all Jomon individuals, we found that a pronounced increase in *N*_e_ occurred approximately 13000–8000 ya, matching the transition from the Incipient to the Initial phase of the Jomon period (Figure 4A). Interestingly, such demographic growth during the Incipient and Initial phase was absent in the BSP of the present-day Japanese individuals, which instead indicated a demographic growth around 5000–2000 ya, corresponding to the Middle to the Final phases of the Jomon period (Figure 4B). This discrepancy likely arises from the present-day Japanese gene pool incorporating mitogenomes from both Jomon people and continental populations who migrated around 3000 ya. Consequently, we infer that demographic growth occurred during the transitional period from the Incipient and Initial phase among the Jomon people but not among continental East Asian populations.

Assuming no gene flow between eastern and western populations, we conducted BSPs for the eastern and western Jomon groups separately. Using the Chubu–Kinki boundary, the coalescent times of eastern and western Jomon did not coincide, making it impossible to observe both demographic plots over the same temporal range (Figure 4C). To address this, we employed the Itoigawa–Shizuoka Tectonic Line, the western edge of Fossa Magna, the central rift valley of the Japanese archipelago, as an alternative boundary. The change of the east–west boundary moved individuals from the Ikawazu and Odake sites, which were previously assigned to the eastern Jomon according to Koyama’s (1978) grouping, into the western Jomon. The reanalysis revealed demographic growth in both the eastern and western Jomon populations, although growth in the Western Jomon population was comparatively moderate (Figure 4D).

### Diversity statistics to test demographic expansion

To elucidate the likely demographic expansion within the Jomon population, we computed diversity statistics using the non-coding regions on mtDNA, which are known to be evolutionarily neutral. Table 2 presents the number of segregation sites (*S*), Watterson’s estimator of population mutation rate (*Θ*_w_), nucleotide diversity (*π*), and Tajima’s *D* for the groups categorized by archaeological site(s) (Iyai, Higashimyo, Odake, and KT GB, SH, combined as Ichihara) and by geographical region(s), comparing them with present-day East Asian populations (CHB and JPT). Among the groups, excluding the present-day populations, the minimum *π* value was 0.00279 (Iyai), while the maximum was 0.00439 (Higashimyo). Both of the values were from the Initial phase of the Jomon period. Eastern and western Jomon exhibited nearly identical *π* values (0.00310 and 0.00314, respectively), indicating comparable genetic diversity between the eastern and western regions of the archipelago. Notably, the *π* value of all Jomon individuals (0.00330) was considerably lower than that of CHB and JPT (0.00884 and 0.00785, respectively), suggesting a relatively reduced genetic diversity among the Jomon people compared to present-day populations.

As for Tajima’s *D*, both all Jomon and present-day Japanese individuals, of which BSPs showed demographic growth at different periods, exhibited significantly negative values, indicating rapid demographic expansion (Table 2). Furthermore, eastern Jomon individuals exhibited significantly negative Tajima’s *D* values, while their western counterparts did not. Thus, it is likely that, overall, the Jomon population experienced a substantial expansion, which is particularly evident in the eastern Jomon population.

## Discussion

In this study, we delved into the demographic history of the Jomon people through comprehensive analyses of whole-mitogenome sequence data involving the Initial, Early, Middle, Late, and Final phases of the Jomon period, from Hokkaido, Hondo, and Ryukyu in the Japanese archipelago (Figure 1, Supplementary Figure S1). Integrating the accumulated Jomon whole-mitogenome sequence data with BSP analysis—a method for estimating overtime changes in *N*_e_ based on haploid DNA phylogeny—enabled us to directly reconstruct the demographics of the Jomon people.

The phylogenetic network constructed from whole-mitogenome sequence data revealed two distinct clades corresponding to M7a and N9b (Figure 2). Notably, a couple of star-like structures were observed exclusively within the N9b clade, suggesting potential signals of demographic expansion. Consistent with previous findings, when Jomon individuals were divided into eastern and western groups using the Chubu–Kinki boundary, the N9b clade predominantly encompassed eastern Jomon individuals, while the M7a clade primarily comprised western Jomon individuals. In previous studies, this east–west frequency difference has been attributed to at least two separate migration waves of Jomon ancestors to the Japanese archipelago: N9b came through a northern route and M7a through a western route (Tanaka et al., 2004; Adachi et al., 2011; Shinoda, 2019). However, our simulation suggests that such a large frequency difference could arise through genetic drift alone, even from a single ancestral population, given a small initial effective population size, an extreme population split, and limited migration between eastern and western Jomon populations (Figure 3). It should be noted that the probability that large haplogroup frequency differences did not occur was higher even in the cases where all of these conditions are met. Our simulation was based on a generalized model that does not incorporate fine-grained assumptions, such as local social structure or marriage system, but instead treats the population at a whole. Although it is well understood that haplogroup frequency differences can arise through genetic drift, this study is, to our knowledge, the first to explicitly integrate multiple lines of empirical evidence for the prehistoric population in the Japanese archipelago—including demographic shifts inferred from the archaeological record, estimated haplogroup divergence time, and the observed east–west frequency differences—into a unified simulation framework. This integrated approach represents a significant advance in the study of Jomon population demography. Discrepancies between model assumptions and archaeological reality, such as the possibility of a population barrier around the Chubu–Kinki boundary, should be further investigated. Such a barrier may have been cultural rather than geographical or environmental in nature (Ono et al., 1992; Harada, 2010; Yamada, 2019). Nevertheless, our findings provide a valuable perspective: the asymmetric distribution of haplogroups N9b and M7a is not necessarily inconsistent with the monophyletic origin of the Jomon people, as suggested by a recent nuclear genome analysis (Watanabe et al., 2024).

The divergence date estimates in this study indicate that haplogroups N9b and M7a emerged approximately 23000 and 26000 ya, respectively (Supplementary Figure S3). These dates are similar to the divergence times estimated from whole-mitogenome sequences of present-day Japanese individuals (Adachi et al., 2011), supporting the hypothesis that both haplogroups in present-day Japanese are of Jomon origin (Horai et al., 1996). Remarkably, these dates fall within the divergence time of the Jomon people from other East Eurasian populations inferred from Jomon nuclear genomes (*n* = 42) (Watanabe et al., 2024). Thus, a population with both haplogroup N9b and M7a at high frequencies may have migrated from East Eurasia to the Japanese archipelago around 25000 ya and become the ancestors of the Jomon people. Additionally, given that both N9b and M7a are rarely found in present-day populations outside the Japanese archipelago, it is possible that these haplogroups underwent substantial diversification within the archipelago itself. To clarify the exact origin of both haplogroups in the future, mtDNA sequences of the Initial Jomon and the Upper Paleolithic individuals in East Eurasia will be necessary.

We observed, in BSP, an increase in mitochondrial *N*_e_ during the Incipient to Initial phase of the Jomon period, a trend that was not shown in the analysis of mitogenome sequences from present-day Japanese populations (Figure 4A, B). This is reasonable because the present-day mitogenomes in the archipelago include those of migrants from the continent at a remarkably high frequency. Notably, the BSP analysis performed on the Y chromosome of present-day Japanese, which is thought to be derived from the Jomon people, showed an increase of *N*_e_ contemporaneous with the period we estimated (Watanabe et al., 2019). *N*_e_ refers to a theoretical value derived from genetic diversity within a population, distinct in concept from the actual population size (see Materials and methods). Increases in *N*_e_ are generally attributable either to rapid internal growth or to the influx of individuals from external populations. In light of the absence of archaeological and genetic evidence for large-scale migration or population replacement in the Japanese archipelago from the Upper Paleolithic to the Jomon period, internal population expansion seems the more plausible scenario. However, the timing of population growth estimated from genome data predates that proposed by Koyama (1978), raising an open question regarding the demographic history of this period. It is possible that archaeological evidence, such as the number of sites, reflects the consequences of the earlier demographic expansion rather than its onset. Alternatively, the difference could stem from uncertainties in our genetic estimates due to parameter choices. Either way, this temporal mismatch deserves further attention as additional data become available.

Archaeological observations have long noted disparities in site numbers between the east and west, with this gap purportedly widening significantly during the Middle phase (Koyama and Sugito, 1984). When using the Chubu–Kinki boundary as the east–west boundary, our results support Koyama’s estimate in terms of the population size, i.e. that of eastern Jomon had been larger than that of western Jomon (Figure 4C). When using the Itoigawa–Shizuoka Tectonic Line, an alternative east–west boundary in this study, more pronounced demographic growth was observed in eastern Jomon than in western Jomon. However, *N*_e_ was consistently larger in western Jomon except for around 10000 ya (Figure 4D). Given that the plots for eastern Jomon were similar to the plot of all Jomon (*n* = 40), regardless of the location of the east–west boundary, the number of western Jomon samples may not be sufficient to perform the regionally separate analysis. In archaeological contexts, efforts have been made to correlate the east–west disparity in site numbers, indicative of population size, with regional differences in natural environments and ecosystems. Eastern Japan, characterized by deciduous broad-leaved forests dominated by beech, contrasts with western Japan’s evergreen broad-leaved forests centered around oak (Taniguchi, 2019). Yamanouchi (1964) proposed the hypothesis that salmon and trout served as crucial animal resources in Eastern Japan. In the next step of this research field, it will be important to interpret Jomon genome information based on these ecological perspectives.

## Data Availability

All whole-mitogenome sequence data determined in this study have been deposited in the DNA DataBank of Japan (DDBJ) (accession number LC805243–LC805255)

## Figures and Tables

**Table 1.** List of ancient and modern human samples

ID	Phase/period	Site	Date range and median (cal BP)	Contamination rate range and median (%)	Average coverage	Haplogroup	Reference
Minato1	Upper Paleolithic	Minatogawa fissure	19900^a^	1.99–7.30; NA	52	M	Mizuno et al., 2021
JpKa6904^b^	Initial Jomon	Kamikuroiwa rock shelter	8991–8646; 8819	NA; 1.46	128	N9b3	Cooke et al., 2021
Iyai1		Iyai rock shelter	8300–8200; NA	0.96–5.58; NA	111	N9b	Mizuno et al., 2020, 2021, 2023
Iyai3			NA	NA	729	M7a	
Iyai4			8300–8200; NA	1.47–2.66; NA	1577	N9b3	
Iyai8			8300–8200; NA	2.72–7.59; NA	100	N9b	
Iyai10			NA	NA	61	N9b	
Iyai12			NA	NA	215	N9b3	
Iyai15			NA	NA	2266	N9b	
Higa002		Higashimyo shell mound	NA	2.50–10.5; NA	42	N9a2a	
Higa006			7934–7792; NA	0.97–9.55; NA	22	M7a1a	
Higa020			NA	0.46–6.70; NA	11	M80”D	
Todo5	Early Jomon	Todoroki shell mound	6210–6094; NA	3.94–11.95; NA	37	M7a1a	
JpOd274^b^		Odake shell mound	6289–6119; 6204	NA; 1.13	74	M7a	Cooke et al., 2021
JpOd6^b^			6179–5934; 6057	NA; 1.55	489	N9b3	
JpOd181^b^			5917–5751; 5836	NA; 0.91	77	N9b1	
JpOd282^b^			5902–5737; 5820	NA; 1.38	41	M7a1	
JpFu1^b^		Funagura shell mound	5590–5478; 5534	NA; 2.15	42	M7a1	Mizuno et al., 2021
Kaso6	Middle Jomon	Kasori shell mound	NA	3.30–7.67; NA	127	M7a	
Uba2		Ubayama shell mound	NA	1.94–6.94; NA	44	N9b	
JpKo2^b^		Kosaku shell mound	4514–4294; 4404	NA; 1.44	87	N9b	Cooke et al., 2021
JpKo13^b^	Late Jomon		3978–3847; 3913	NA; 1.5	72	N9b1	
JpHi01^b^		Hirajo shell mound	3850–3685; 3768	NA; 1.45	43	M7a1a	
**SH7-2**		Saihiro shell mound	4231–4088; 4141	0.56–1.49; 1.02	128	N9b1	This study
**SH7-3**			4152–4004; 4102	0.60–2.52; 1.56	26	N9b1	
**SH7-4**			4089–3981; 4036	–0.09–0.50; 0.21	20	N9b	
**SH7-6**			3969–3863; 3912	0.82–1.64; 1.23	268	N9b1a	
**SH7-7**			4356–4185; 4279	0.35–1.26; 0.80	113	N9b3	
**SH7-12**			4225–4080; 4127	0.79–2.84; 1.81	130	M7a2a	
**GB3-3**		Gionbara shell mound	3697–3623; 3664	0.94–1.96; 1.45	123	N9b	
**GB3-9**			3982–3901; 3951	0.11–2.59; 1.35	33	N9b3	
**GB4-S022**			4231–4096; 4169	0.41–0.93; 0.67	191	N9b1a	
**KT2**		Kikumatenaga shell mound	3678–3514; 3589	0.21–0.88; 0.54	80	N9b3	
**KT52-2**			4145–3998; 4076	0.46–1.77; 1.12	102	M7a2a	
**KT68**			3956–3837; 3887	0.35–1.14; 0.74	117	N9b1	
**KT69**			3821–3688; 3736	0.95–2.03; 1.49	161	N9b3	
MB-TB27		Mabuni hantabaru	NA	–1.58–6.58; NA	15	M7a1a	Mizuno et al., 2021
F5^b^		Funadomari	NA	2.04–2.75; 2.45	206	N9b1	Kanzawa-Kiriyama et al., 2019
F23^b^			3846–3644; 3755	1.05–1.33; 1.20	290	N9b1	
IK001	Final Jomon	Ikawazu shell mound	2699–2367; NA	NA	258	M7a1	Waku et al., 2022
IK002^c^			2720–2418; 2569	0.01–2.2; 0.50	146	N9b1	McColl et al., 2018
JN655840.1	Modern	Sudan	Present-day	-	-	L3	Soares et al., 2012

NA, not available. The names of newly reported individuals are indicated in bold. Their date information are referred from Nakamura et al. (2024). ^a^ The currently reported date of Minato 1. ^b^ FASTQ-formatted read data was obtained. ^c^ BAM-formatted read data was obtained.

**Table 2.** Statistical summary of the Jomon groups

Group/site	Phase/period	*S*	*Θ*w	*π*	SD of *π*	Tajima’s *D*
Iyai (*n* = 7)	Initial Jomon	10	0.00376	0.00279	0.00091	–1.35933
Higashimyo (*n* = 3)		7	0.00441	0.00439	0.00122	NA
Odake (*n* = 4)	Early Jomon	6	0.00301	0.00305	0.00090	0.17969
Kikumatenaga (*n* = 4)	Late Jomon	6	0.00301	0.00305	0.00111	0.17969
Gionbara (*n* = 3)		4	0.00245	0.00244	0.00091	NA
Saihiro (*n* = 6)		9	0.00363	0.00293	0.00111	–1.12062
Ichihara (*n* = 13)		13	0.00387	0.00286	0.00074	–1.04304
Eastern Jomon (*n* = 26)	Initial to Late Jomon	27	0.00664	0.00310	0.00057	–1.93676*
Western Jomon (*n* = 14)	Initial to Final Jomon	15	0.00455	0.00314	0.00038	–1.25619
All (*n* = 40)	Initial to Final Jomon	31	0.00712	0.00330	0.00041	–1.82472*
CHB (*n* = 103)	Modern	127	0.02389	0.00884	0.00031	–2.08002*
JPT (*n* = 104)		111	0.02066	0.00785	0.00037	–1.99075*

*S* is the number of segregating sites, ***Θ*_w_** = *p*_s_/*a*_1_ (*p*_s_ = *S*/*n*, *a*_1_ = Σ [*i* = 1 → *n* – 1] 1/*i*), and *π* is the nucleotide diversity. Statistically significant values (*P < 0.05) are indicated in bold with asterisk. NA, not available (because of the small sample size). Ichihara (*n* = 13) includes the KT, GB, and SH sites. CHB, modern Chinese from Beijing; JPT, modern Japanese from Tokyo.

## Supplementary Material

Supplementary Figure S2_10

Supplementary Figure S3A

Supplementary Figure S3B
