# Supplementary figures and images for: Demographic history of the Jomon people: insights from whole-mitogenome analysis
Source: Anthropol Sci. 2026 Jan 30;134(1):15–28. doi: 10.1537/ase.251024 (PMC13134050; doi:10.1537/ase.251024)

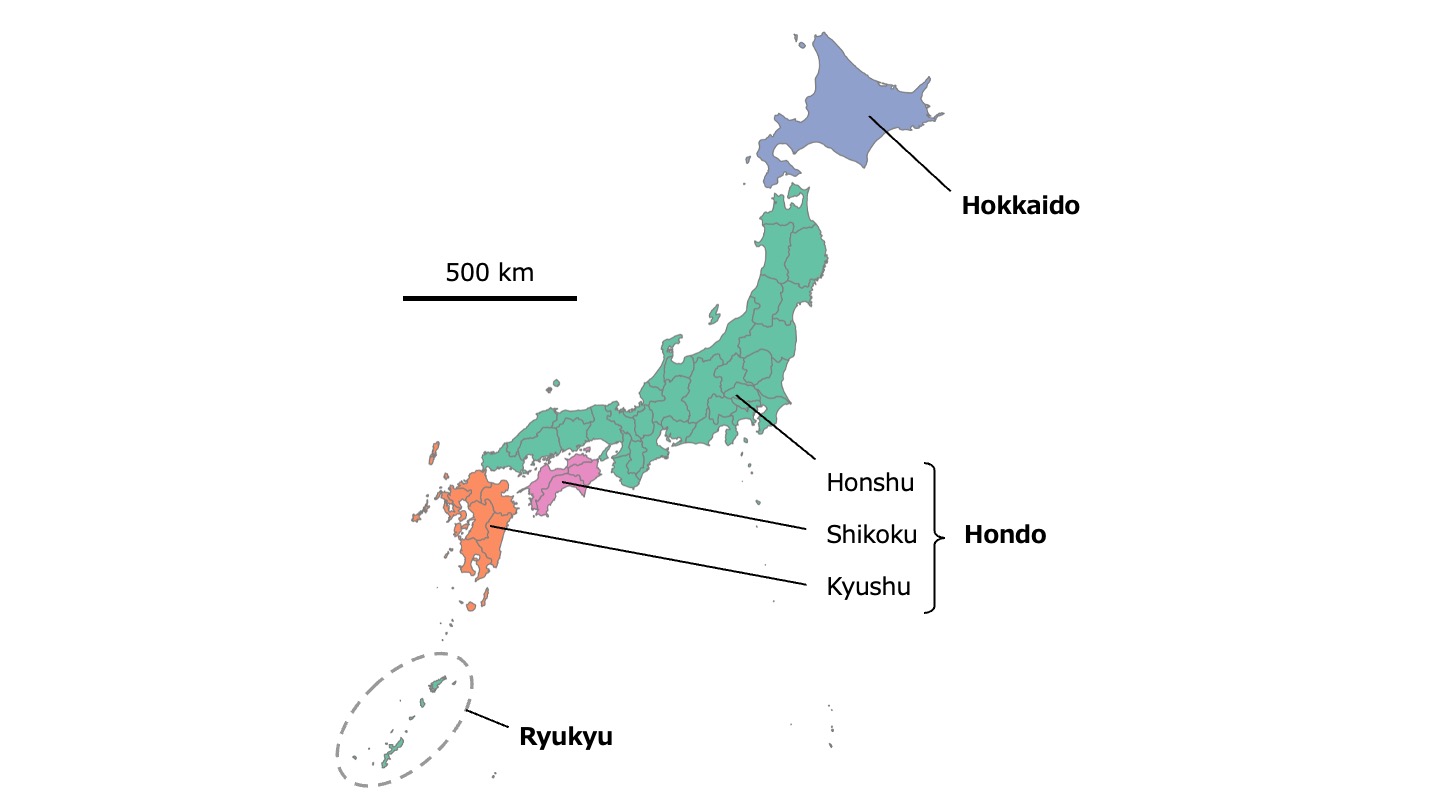

Supplement: Supplementary file 1 — Supplementary Figure S1 [file 134_251024_1.jpg]

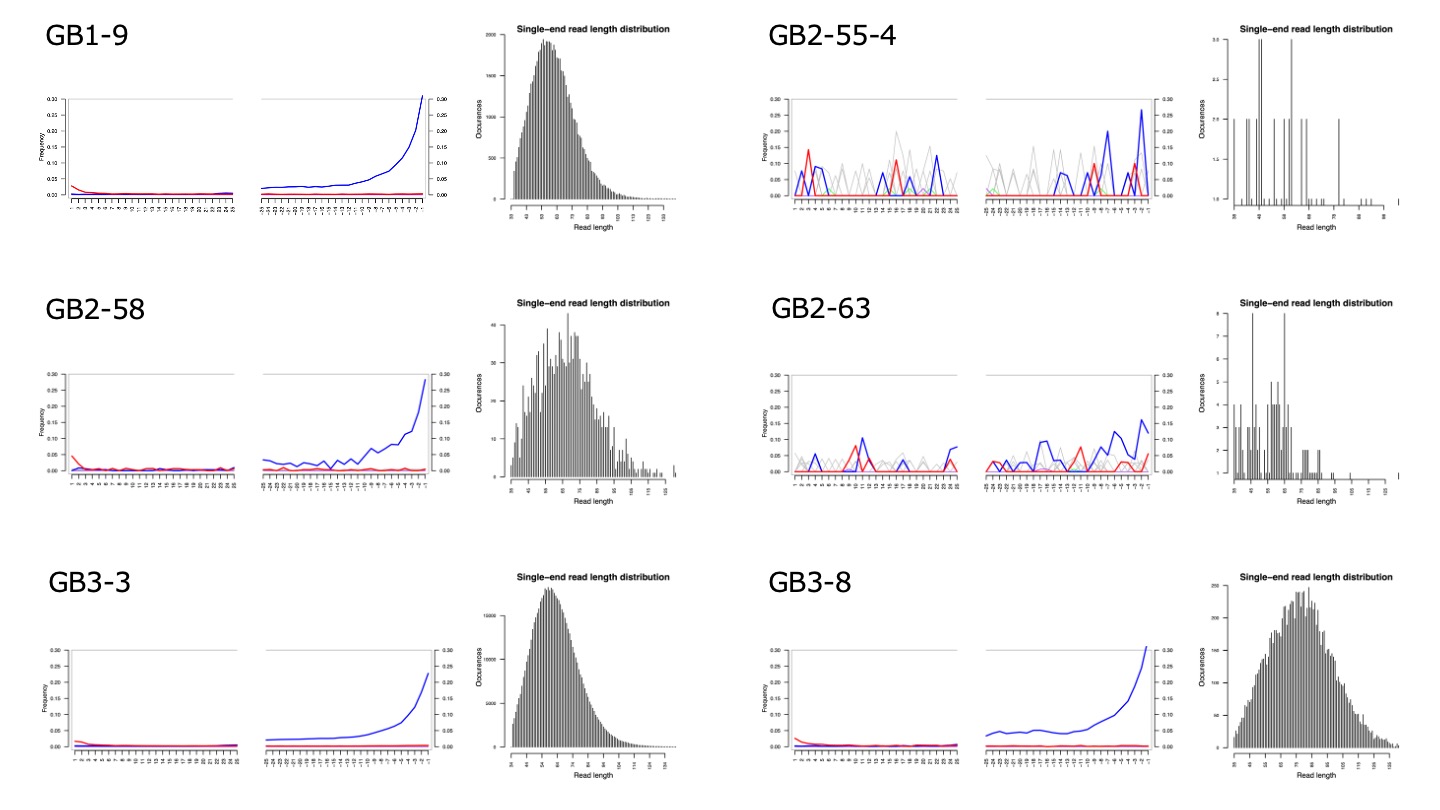

Supplement: Supplementary file 2 — Supplementary Figure S2_1 [file 134_251024_2.jpg]

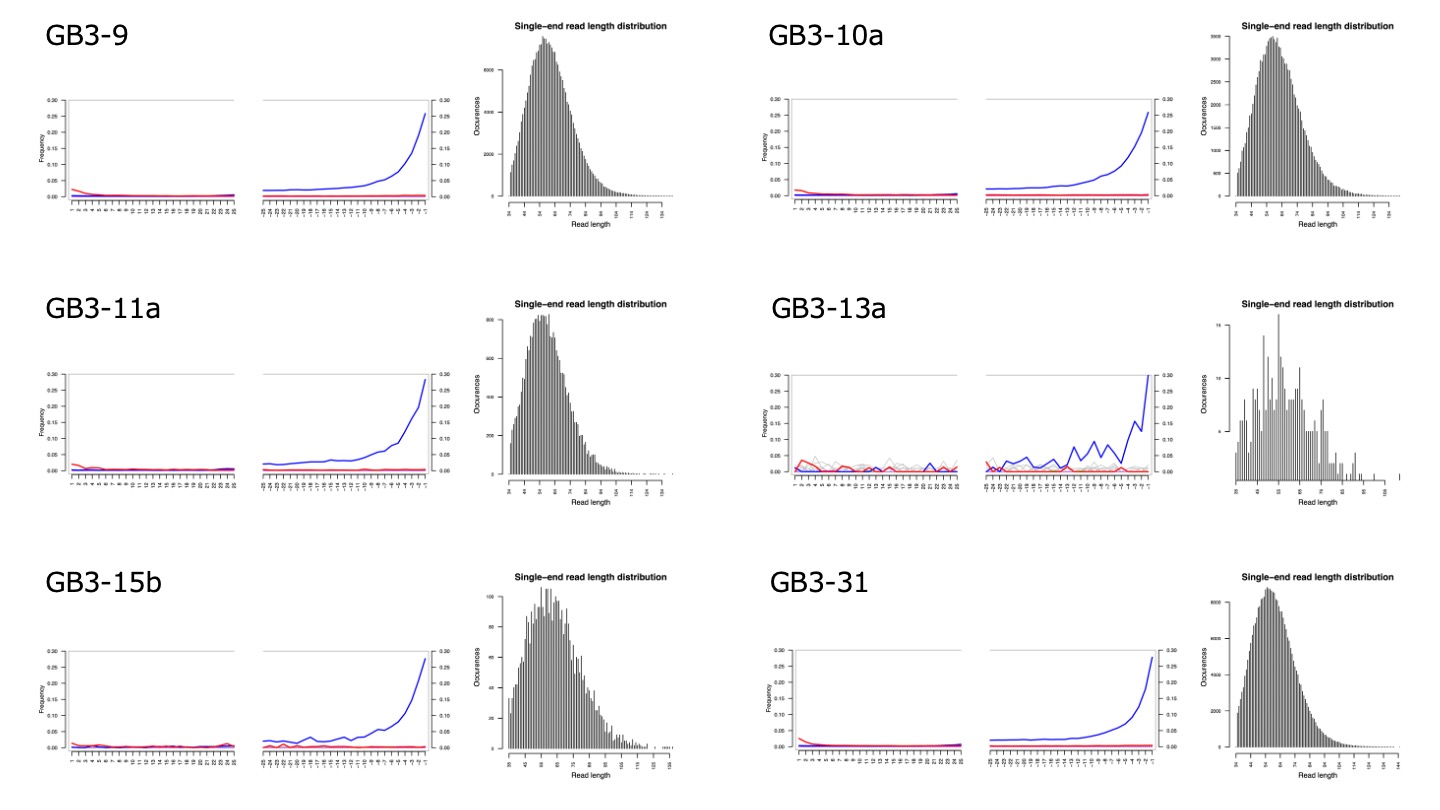

Supplement: Supplementary file 3 — Supplementary Figure S2_2 [file 134_251024_3.jpg]

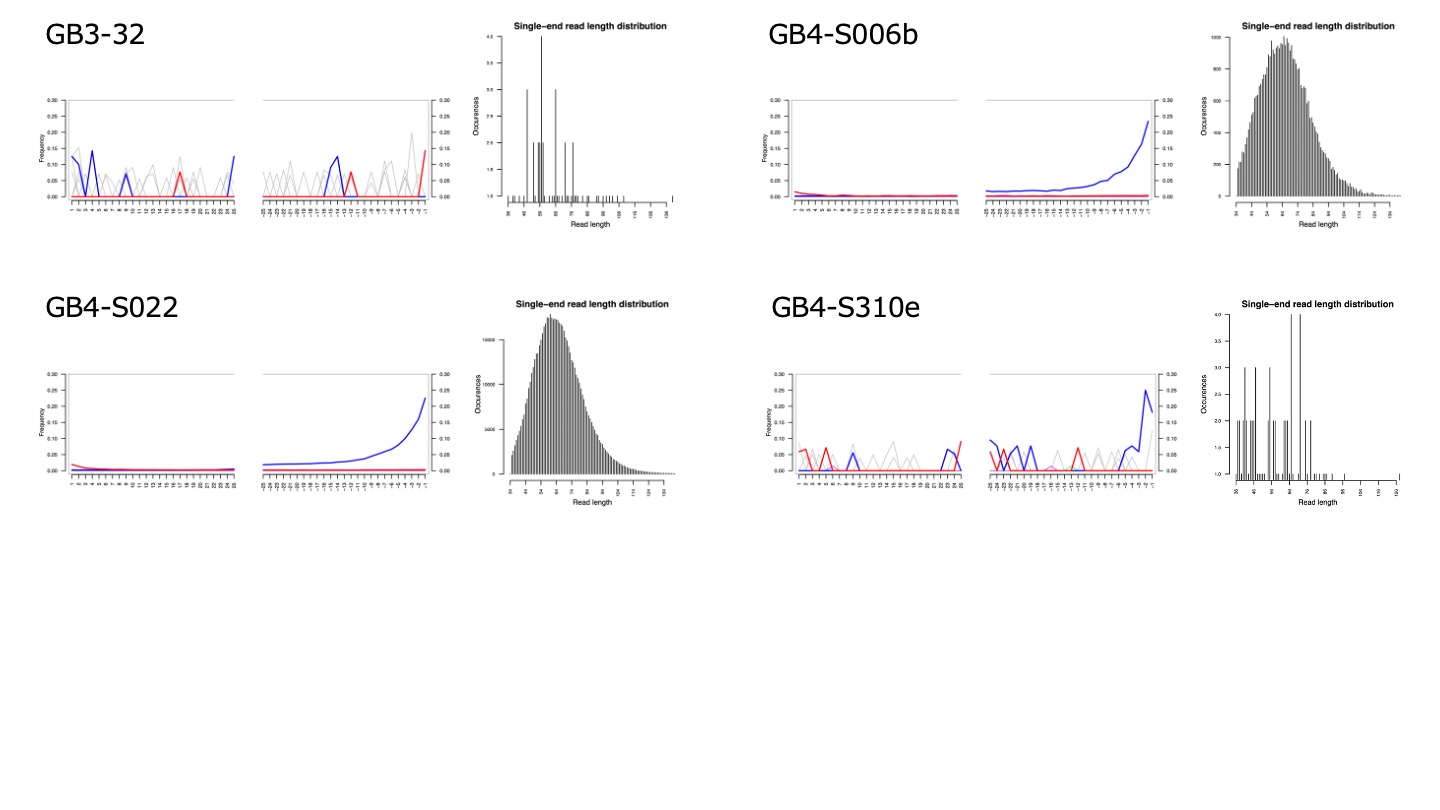

Supplement: Supplementary file 4 — Supplementary Figure S2_3 [file 134_251024_4.jpg]

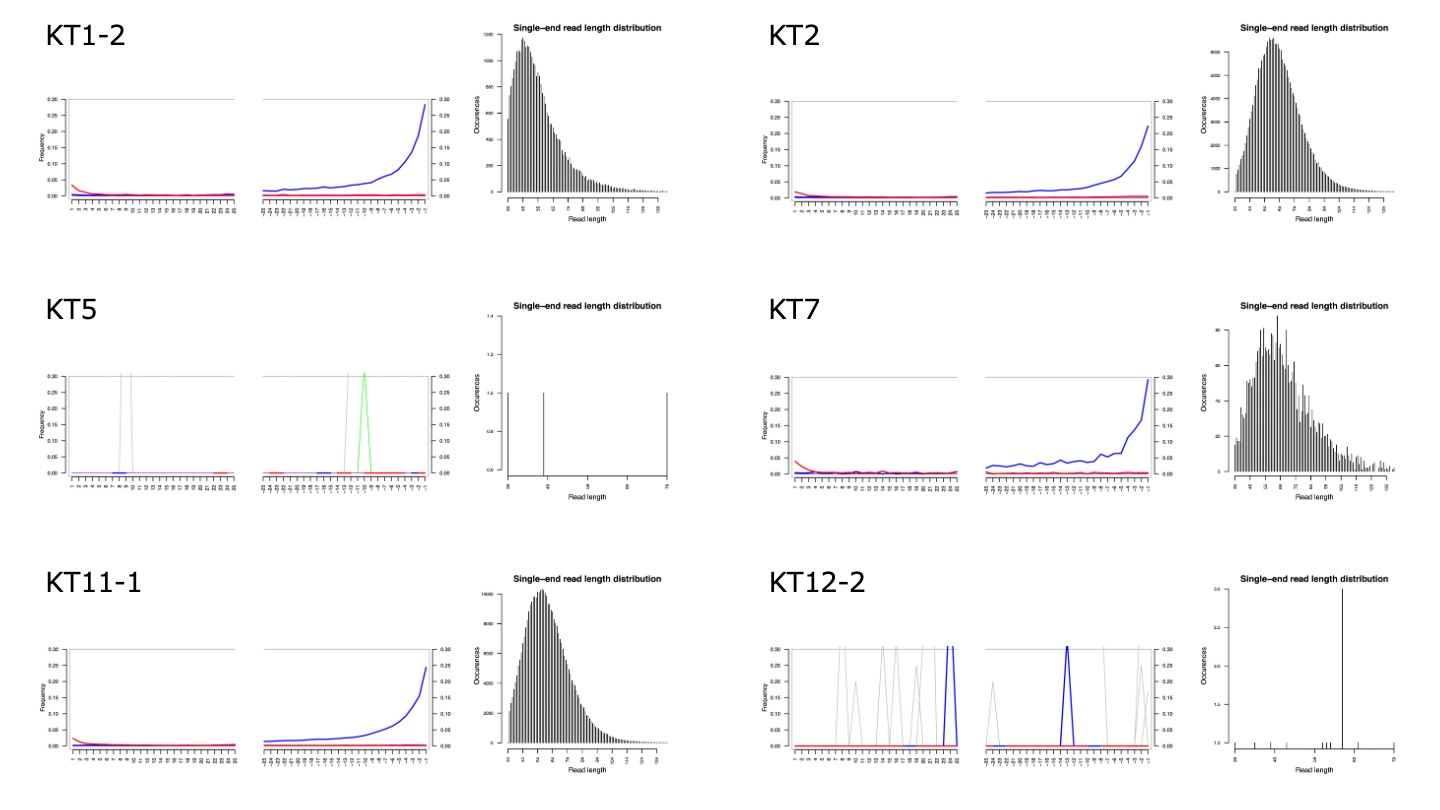

Supplement: Supplementary file 5 — Supplementary Figure S2_4 [file 134_251024_5.jpg]

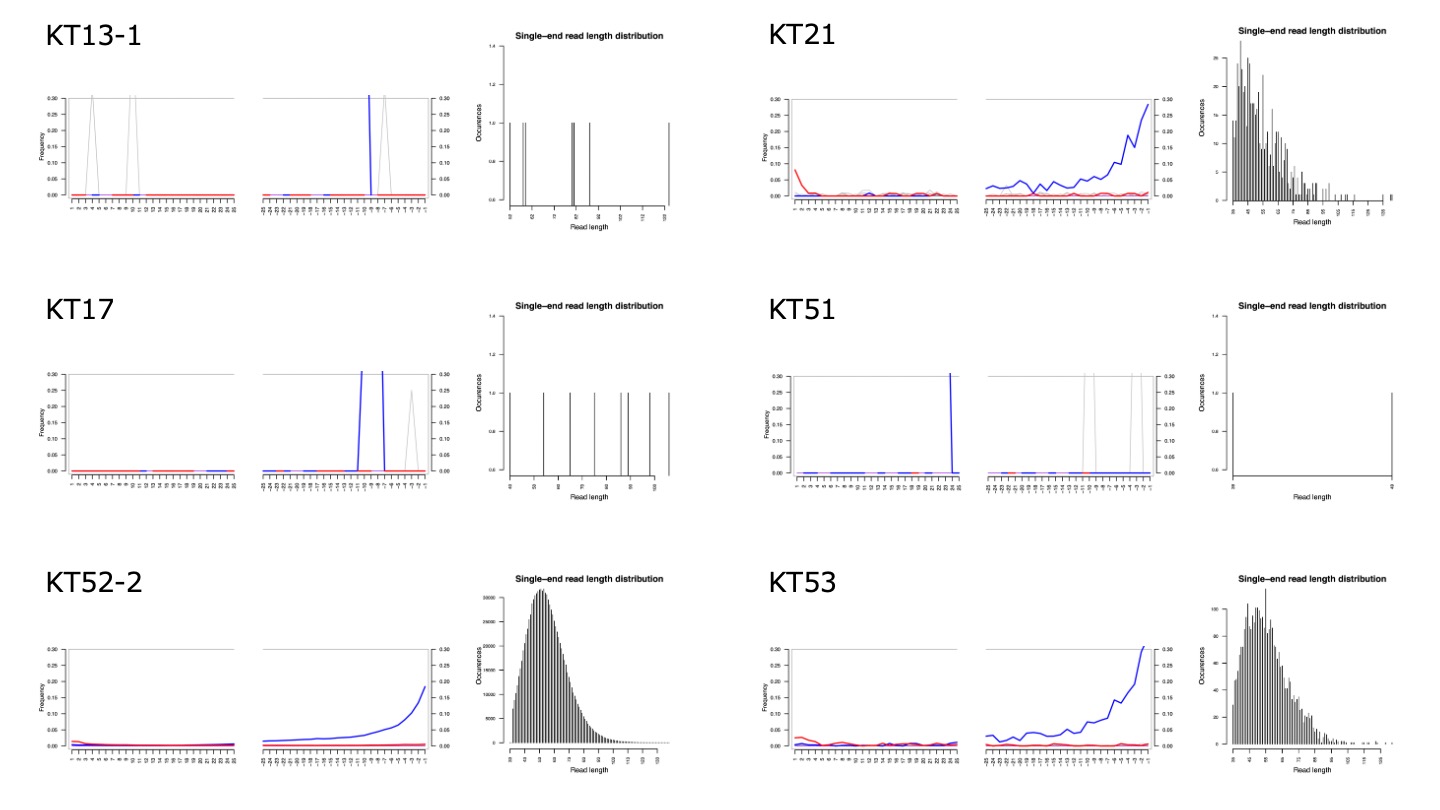

Supplement: Supplementary file 6 — Supplementary Figure S2_5 [file 134_251024_6.jpg]

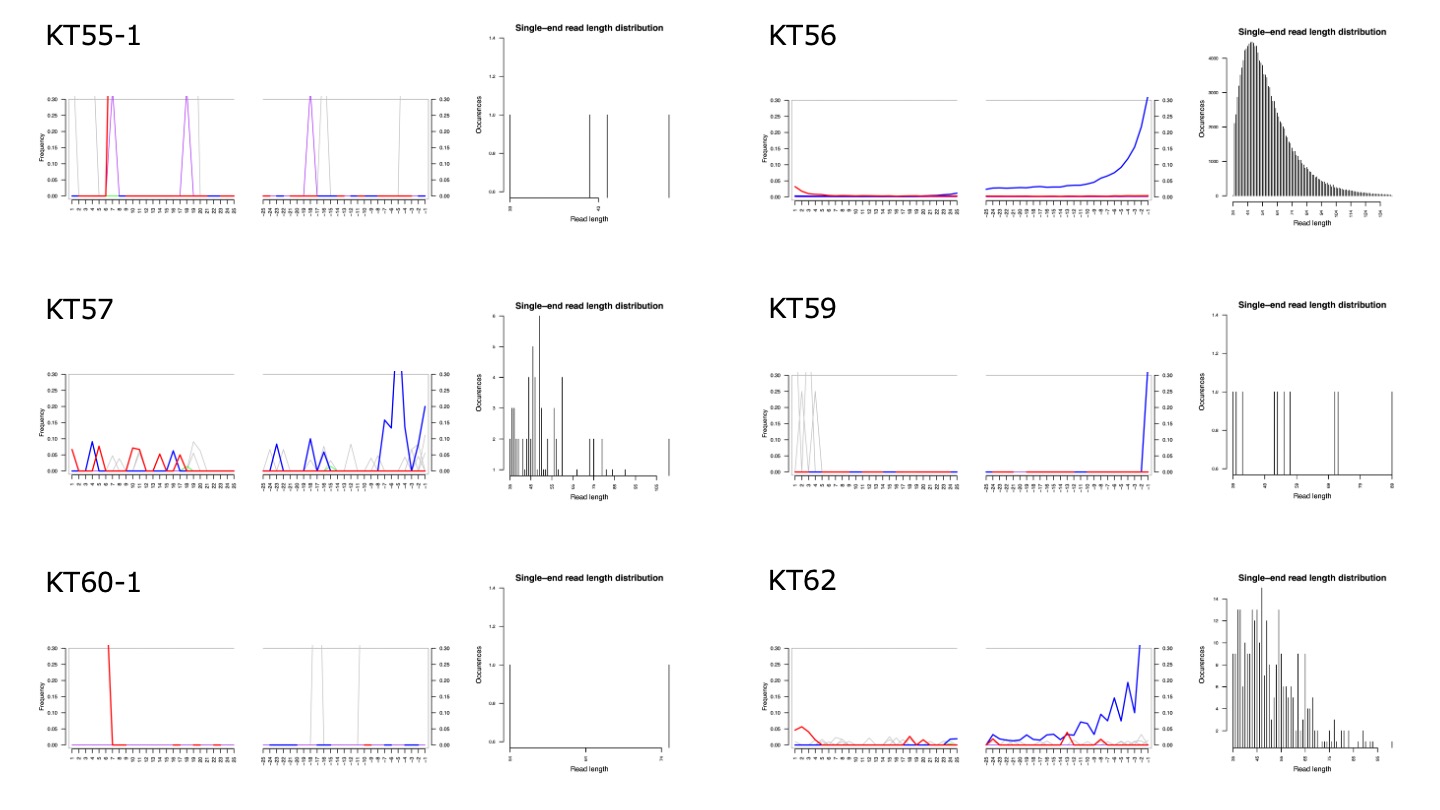

Supplement: Supplementary file 7 — Supplementary Figure S2_6 [file 134_251024_7.jpg]

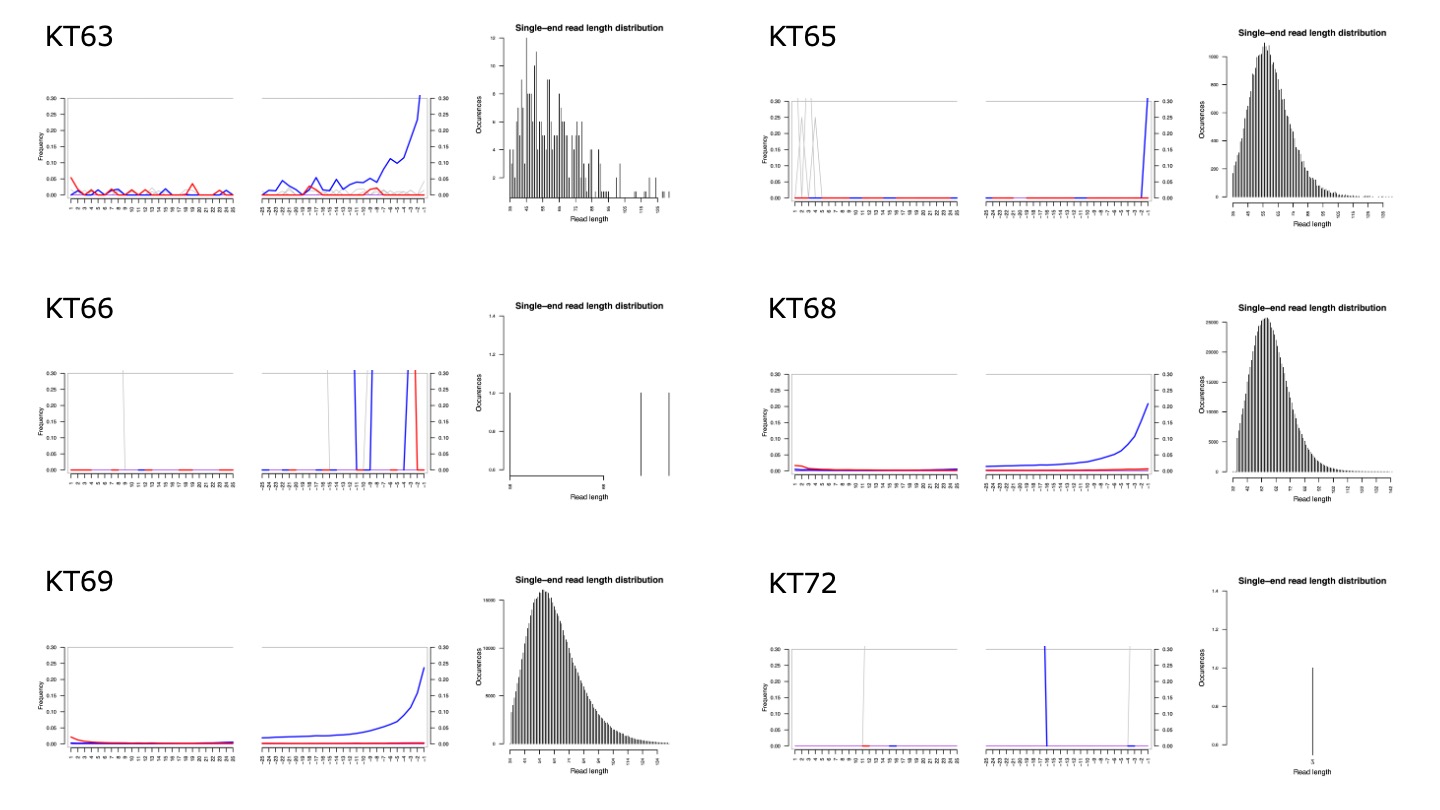

Supplement: Supplementary file 8 — Supplementary Figure S2_7 [file 134_251024_8.jpg]

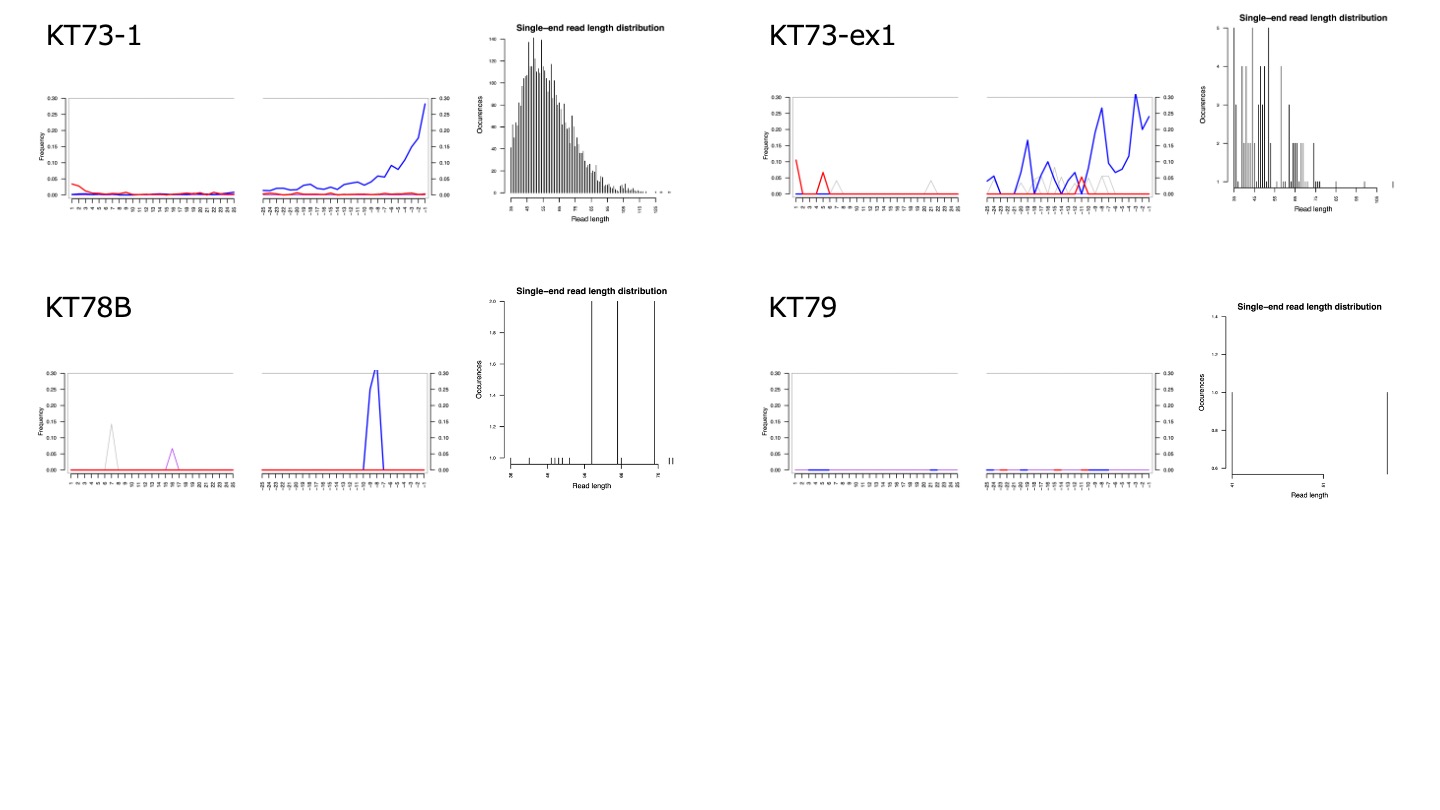

Supplement: Supplementary file 9 — Supplementary Figure S2_8 [file 134_251024_9.jpg]

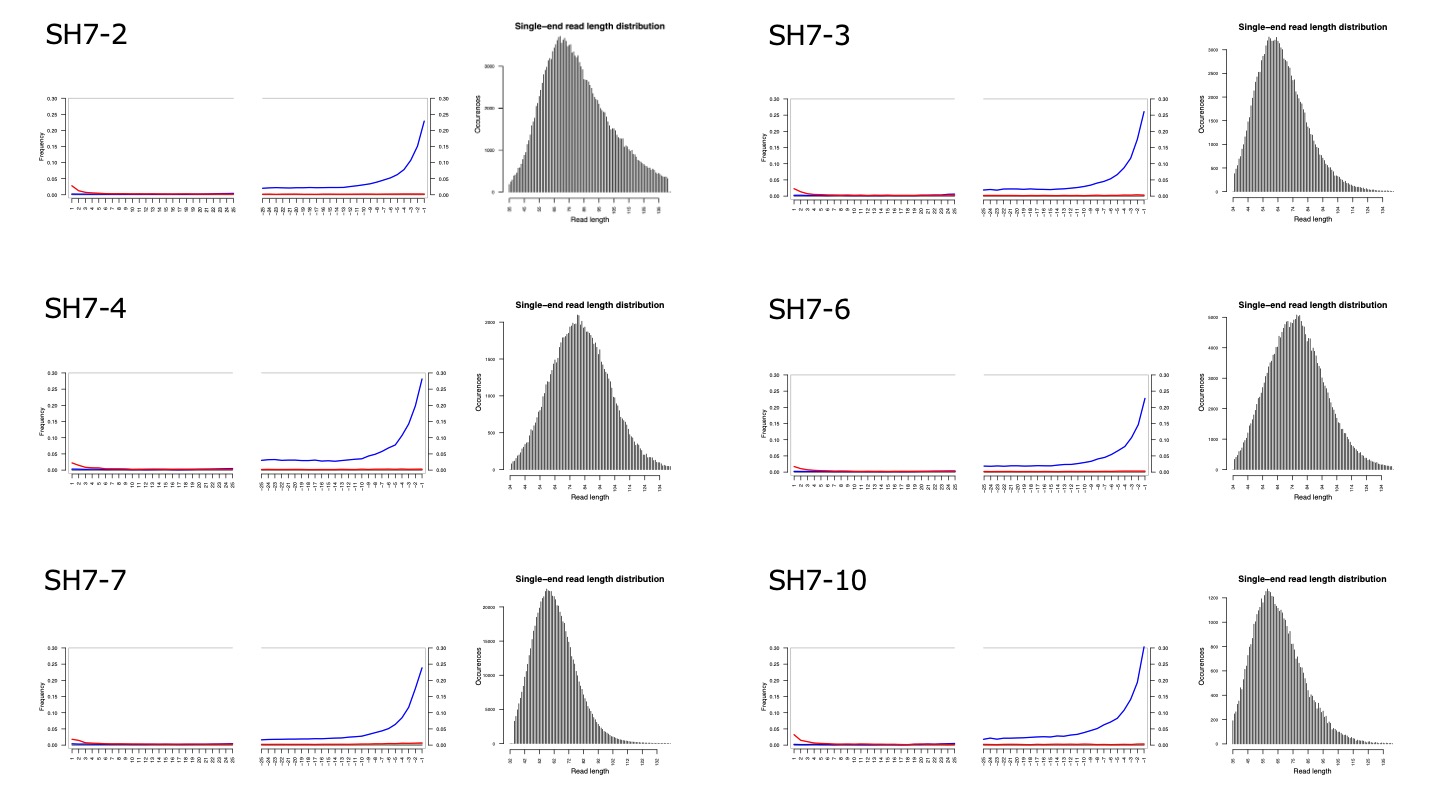

Supplement: Supplementary file 10 — Supplementary Figure S2_9 [file 134_251024_10.jpg]

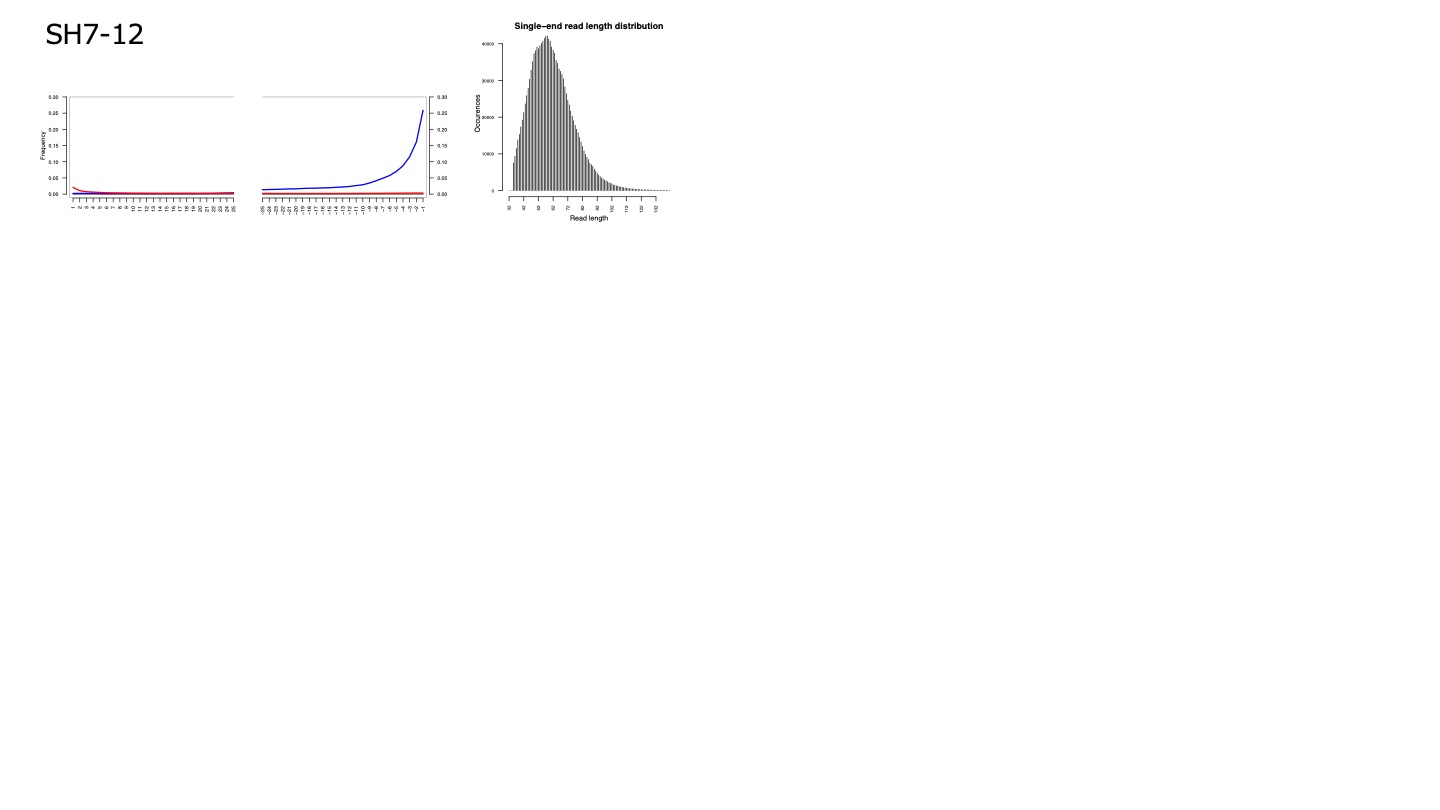

Supplement: Supplementary file 11 — Supplementary Figure S2_10 [file 134_251024_11.jpg]

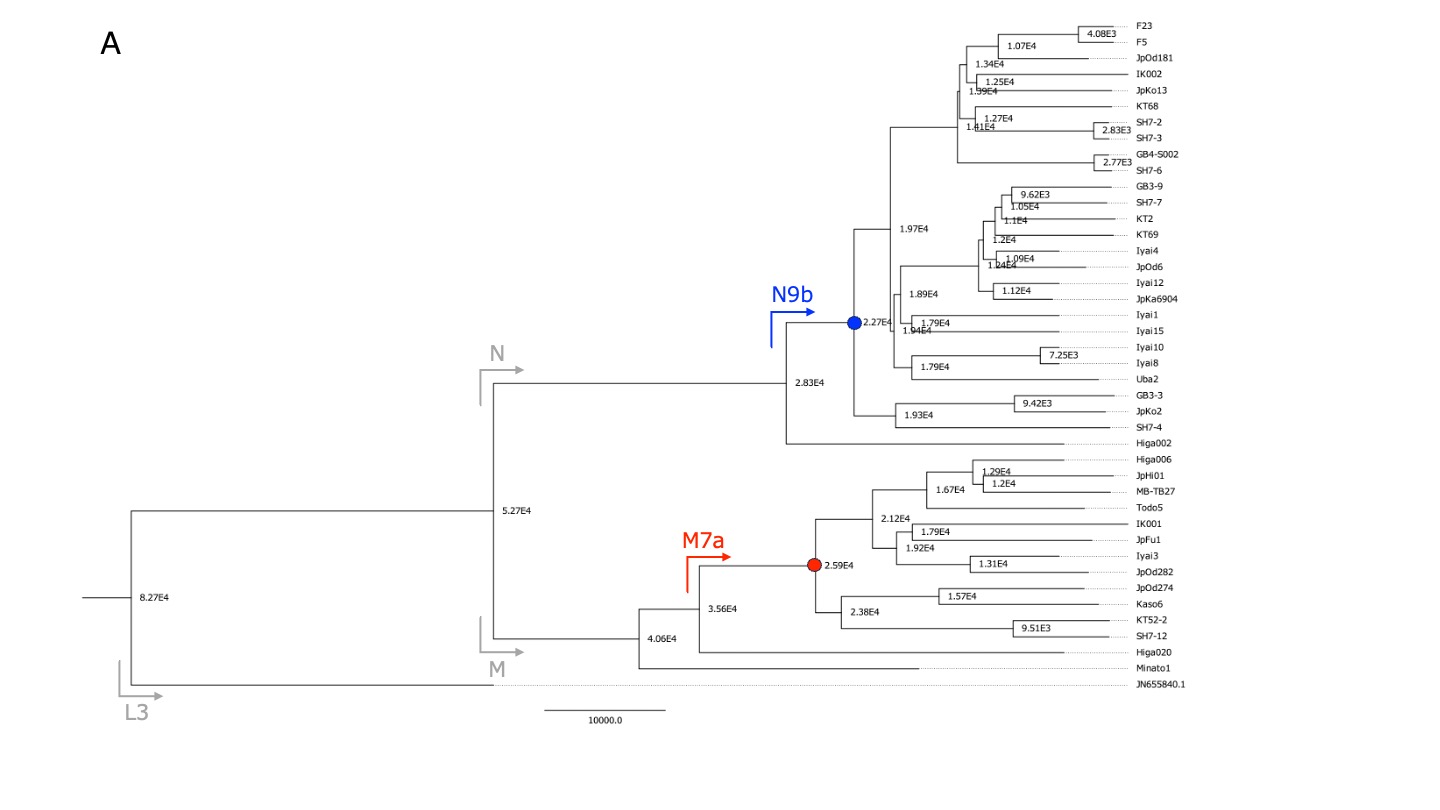

Supplement: Supplementary file 12 — Supplementary Figure S3A [file 134_251024_12.jpg]

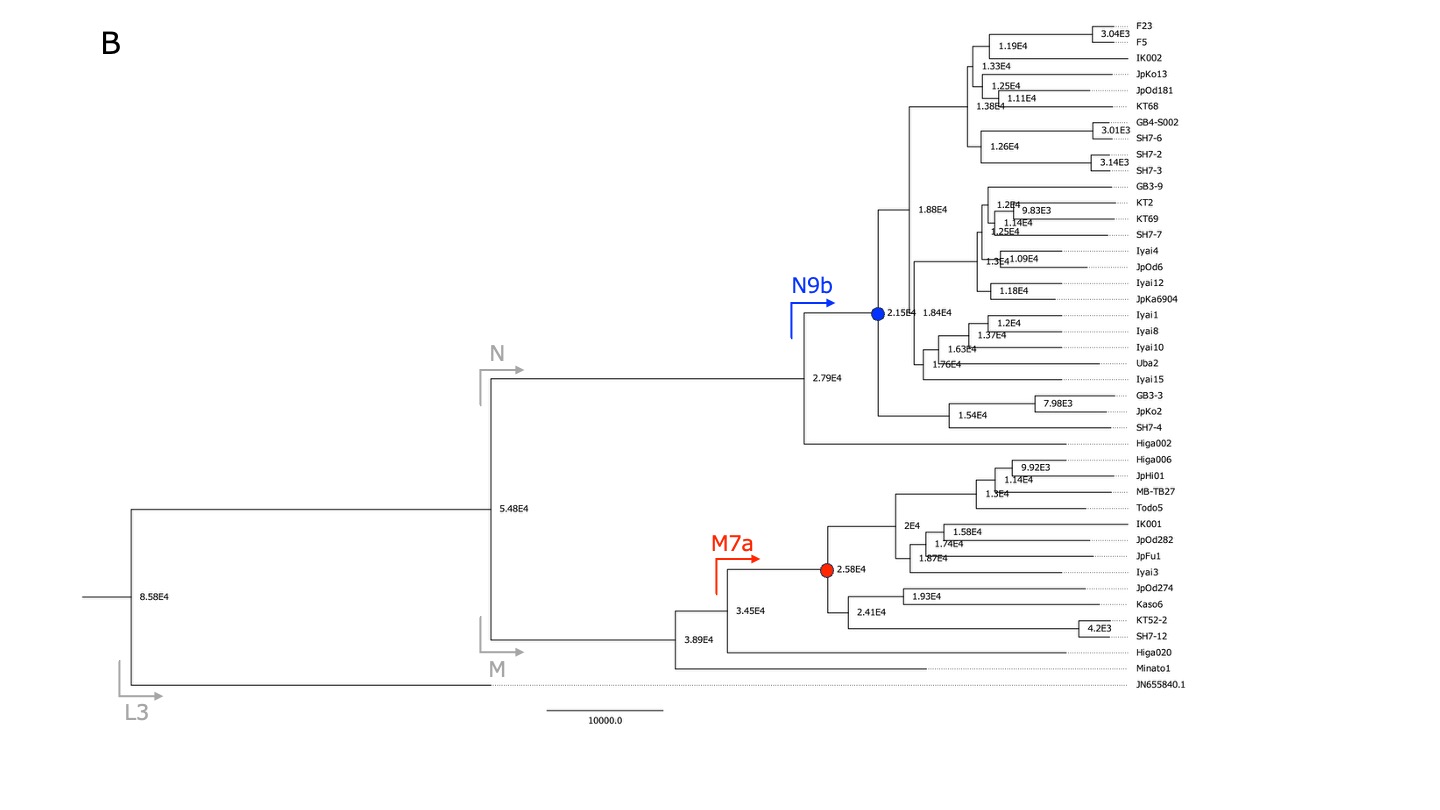

Supplement: Supplementary file 13 — Supplementary Figure S3B [file 134_251024_13.jpg]
