# Supplementary material for: Demographic history of the Jomon people: insights from whole-mitogenome analysis
Source: Anthropol Sci. 2026 Jan 30;134(1):15–28. doi: 10.1537/ase.251024 (PMC13134050; doi:10.1537/ase.251024)
Supplement: Supplementary file 14 — Table S1 [file 134_251024_14.pdf]

Table S1. Summary of ancient samples

Sample ID	Shell mound	Skeletal part	DNA conc. (ng/μl)	Mapping rate (%)	^{14}C date (BP)	Calibrated date 1σ (cal BP)	Calibrated date Median (cal BP)	AMS-ID	Shell stratum	Reference
GB1-9	Gionbara	Petrous	3.16	2.43	3548±21	3883 (57.9%) 3828<br>3788 (10.3%) 3775	3843	TKA-22956	Out	Nakamura *et al*., 2024
GB2-A1a	Gionbara	Petrous	N.A.	N.A.	N.A.	N.A.	N.A.	N.A.	N.A.	This study
GB2-55-4	Gionbara	Petrous	2.10	0.02	N.A.	N.A.	N.A.	N.A.	Out	This study
GB2-58	Gionbara	Petrous	2.20	0.28	3984±24	4385 (6.6%) 4370<br>4357 (61.7%) 4242	4304	TKA-22954	Out	Nakamura *et al*., 2024
GB2-63	Gionbara	Petrous	2.55	0.01	N.A.	N.A.	N.A.	N.A.	Out	Nakamura *et al*., 2024
GB3-3	Gionbara	Petrous	1.04	**44.52**	3573±21	3623 (68.3%) 3697	3664	TKA-22962	In	Nakamura *et al*., 2024
GB3-8	Gionbara	Petrous	1.97	2.01	3779±23	3823 (22.9%) 3785<br>3780 (45.4%) 3705	3933	TKA-22975	In	Nakamura *et al*., 2024
GB3-9	Gionbara	Petrous	0.53	**18.66**	3738±23	3901 (68.3%) 3982	3951	TKA-22990	Out	Nakamura *et al*., 2024
GB3-10a	Gionbara	Petrous	0.59	7.20	3764±23	3720 (63.1%) 3614<br>3604 (5.1%) 3591	3668	TKA-23031	Out	Nakamura *et al*., 2024
GB3-11a	Gionbara	Petrous	0.60	1.55	3643±22	3979 (68.3%) 3870	3752	TKA-22982	In	Nakamura *et al*., 2024
GB3-13a	Gionbara	Petrous	0.80	0.02	3907±21	3964 (12.2%) 3945<br>3930 (56.1%) 3851	4133	TKA-22949	In	Nakamura *et al*., 2024
GB3-15b	Gionbara	Petrous	3.08	0.79	3420±23	3567 (68.3%) 3485	3529	TKA-22989	N.A.	Nakamura *et al*., 2024
GB3-31	Gionbara	Petrous	2.32	9.52	3935±26	4239 (57.9%) 4142<br>4120 (10.4%) 4098	4182	TKA-22992	Out	Nakamura *et al*., 2024
GB3-32	Gionbara	Petrous	3.34	0.01	N.A.	N.A.	N.A.	N.A.	Out	Nakamura *et al*., 2024
GB4-310o	Gionbara	Petrous	2.57	0.01	3824±27	4087 (68.3%) 3977	4030	TKA-22967	Out	Nakamura *et al*., 2024
GB4-S006b	Gionbara	Petrous	2.14	5.81	3651±22	3909 (68.3%) 3830	3869	TKA-22978	Out	Nakamura *et al*., 2024
GB4-S022	Gionbara	Petrous	0.83	**30.46**	3864±24	4231 (24.0%) 4197<br>4185 (27.7%) 4144<br>4121 (16.6%) 4096	4169	TKA-20259	Out	Nakamura *et al*., 2024
KT1-2	Kikumatenaga	Petrous	0.47	11.90	3738±22	3964 (12.1%) 3945<br>3930 (56.1%) 3851	3905	TKA-25370	N.A.	Nakamura *et al*., 2024
KT2	Kikumatenaga	Petrous	0.11	**36.20**	3444±21	3964 (12.1%) 3945<br>3930 (56.1%) 3851	3589	TKA-25368	N.A.	Nakamura *et al*., 2024
KT5	Kikumatenaga	Tooth	0.60	0.01	3602±20	N.A.	N.A.	TKA-25384	N.A.	This study
KT7	Kikumatenaga	Petrous	0.37	8.79	3544±21	N.A.	N.A.	TKA-24752	N.A.	This study
KT11-1	Kikumatenaga	Petrous	0.34	13.93	N.A.	N.A.	N.A.	N.A.	N.A.	Nakamura *et al*., 2024
KT12-2	Kikumatenaga	Molar	0.27	0.05	N.A.	N.A.	N.A.	N.A.	N.A.	This study

KT13-1	Kikumatenaga	Petrous	0.60	0.01	4093±22	"4515 (26.9%) 4468<br>4450 (41.3%) 4394"	4429	TKA-25314	N.A.	Nakamura *et al*., 2024
KT17	Kikumatenaga	Molar	0.17	N.A.	3885±20	N.A.	N.A.	TKA-24739	N.A.	This study
KT21	Kikumatenaga	Petrous	0.06	8.77	N.A.	N.A.	N.A.	N.A.	N.A.	Nakamura *et al*., 2024
KT51	Kikumatenaga	Molar	0.25	0.00	N.A.	N.A.	N.A.	N.A.	N.A.	This study
KT52-2	Kikumatenaga	Petrous	0.10	**58.13**	3897±21	4145 (45.0%) 4063<br>4042 (23.3%) 3998	4076	TKA-24758	N.A.	Nakamura *et al*., 2024
KT53	Kikumatenaga	Petrous	0.13	1.01	3706±21	3846 (68.3%) 3725	3793	TKA-25378	N.A.	Nakamura *et al*., 2024
KT55-1	Kikumatenaga	Molar	0.58	0.01	3932±21	N.A.	N.A.	TKA-24761	N.A.	This study
KT56	Kikumatenaga	Petrous	1.45	2.99	3786±22	N.A.	N.A.	TKA-24759	N.A.	This study
KT57	Kikumatenaga	Petrous	0.04	0.03	3723±21	3956 (2.1%) 3952<br>3926 (66.1%) 3837	3887	TKA-25377	N.A.	Nakamura *et al*., 2024
KT59	Kikumatenaga	Petrous	0.09	0.02	3649±21	N.A.	N.A.	TKA-25388	N.A.	This study
KT60-1	Kikumatenaga	Molar	0.28	0.01	N.A.	N.A.	N.A.	N.A.	N.A.	This study
KT62	Kikumatenaga	Petrous	0.54	0.95	3760 ± 21	3985 (68.3%) 3890	3944	TKA-25380	N.A.	Nakamura *et al*., 2024
KT63	Kikumatenaga	Petrous	0.73	0.40	3637 ± 21	3822 (21.0%) 3788<br>3777 (47.3%) 3703	3757	TKA-25381	N.A.	Nakamura *et al*., 2024
KT65	Kikumatenaga	Petrous	0.45	1.96	3708 ± 21	3925 (68.3%) 3837	3886	TKA-25376	N.A.	Nakamura *et al*., 2024
KT66	Kikumatenaga	Molar	0.33	0.01	3749 ± 22	N.A.	N.A.	TKA-25371	N.A.	This study
KT68	Kikumatenaga	Petrous	0.13	**56.22**	3723 ± 22	3956 (2.1%) 3952<br>3926 (66.1%) 3837	3887	TKA-25373	N.A.	Nakamura *et al*. 2024
KT69	Kikumatenaga	Petrous	0.10	**62.97**	3615 ± 22	3821 (17.0%) 3790<br>3774 (51.2%) 3688	3736	TKA-25374	N.A.	Nakamura *et al*. 2024
KT72	Kikumatenaga	Premolars	0.35	0.00	N.A.	N.A.	N.A.	N.A.	N.A.	This study
KT73-1	Kikumatenaga	Petrous	0.12	12.56	N.A.	N.A.	N.A.	N.A.	N.A.	This study
KT73-ex1	Kikumatenaga	Molar	0.18	0.23	N.A.	N.A.	N.A.	N.A.	N.A.	This study
KT78B	Kikumatenaga	Molar	0.12	0.04	N.A.	N.A.	N.A.	N.A.	N.A.	This study
KT79	Kikumatenaga	Molar	0.08	0.01	3736±22	N.A.	N.A.	TKA-25365	N.A.	This study
SH7-2	Saihiro	Petrous	1.63	**30.84**	3851±27	4227 (14.5%) 4201<br>4180 (5.1%) 4169<br>4160 (48.7%) 4088	4141	TKA-20252	N.A.	Nakamura *et al*., 2024
SH7-3	Saihiro	Petrous	2.16	**19.65**	3837±24	4152 (53.3%) 4076<br>4035 (15.0%) 4004	4102	TKA-20253	N.A.	Nakamura *et al*., 2024
SH7-4	Saihiro	Petrous	0.81	14.94[†]	3838±24	4089 (68.3%) 3981	4036	TKA-20254	N.A.	Nakamura *et al*., 2024
SH7-6	Saihiro	Petrous	1.42	**36.24**	3747±25	3969 (68.3%) 3863	3912	TKA-20255	Out	Nakamura *et al*., 2024
SH7-7	Saihiro	Petrous	0.99	**48.64**	3999±25	4356 (63.9%) 4227<br>4197 (4.4%) 4185	4279	TKA-20256	N.A.	Nakamura *et al*., 2024

SH7-10	Saihiro	Petrous	1.83	9.03	3302±25	3455 (68.3%) 3393	3424	TKA-20257	N.A.	Nakamura *et al* ., 2024
SH7-12	Saihiro	Petrous	1.37	**42.04**	3910±26	4225 (10.0%) 4200<br>4182 (58.3%) 4080	4127	TKA-20258	N.A.	Nakamura *et al* ., 2024

Mapping ratios >15% are indicated in bold type.

N.A. represents "not available" data.

† The mapping ratio did not exceed 15% but was almost 15%, so subsequent sequencing was performed.
